# The assembly of tetrameric human L‐lactate dehydrogenase is regulated by ionic strength, β‐NADH binding, cyclic peptides, and long‐chain dicarboxylates

**DOI:** 10.1002/pro.70796

**Published:** 2026-09-18

**Authors:** Alessandra Stefan, Luca Gentilucci, Alberto Giuseppe Barbiroli, Valentina Rossi, Hang Liao, Giuseppina Di Stefano, Tingting He, Alejandro Hochkoeppler

**Affiliations:** ^1^ Department of Pharmacy and Biotechnology University of Bologna Bologna Italy; ^2^ CSGI University of Florence Florence Italy; ^3^ Department of Chemistry “Giacomo Ciamician” University of Bologna Bologna Italy; ^4^ Department of Food, Environmental and Nutritional Sciences University of Milan Milan Italy; ^5^ Department of Medical and Surgical Sciences University of Bologna Bologna Italy

**Keywords:** assembly, human lactate dehydrogenase, inhibitors, monomer

## Abstract

Lactate dehydrogenases catalyze the reduction of pyruvate to lactate, the generation of which is of importance for the energetic metabolism of malignant cells. In particular, human cancer cells overexpress lactate dehydrogenase A (hLDH‐A), whose catalytic activity depends on its assembly into the corresponding homotetramer (denoted as hLDH‐5), suggesting that compounds interfering with the protein–protein interactions responsible for the generation of hLDH‐5 should represent inhibitors of high selectivity. Not surprisingly, quite a number of competitive inhibitors were designed, synthesized, and tested against hLDH‐5. However, only a few representatives of the repertoire accordingly obtained feature an appropriate selectivity. Here we show how the ionic strength and β‐NADH binding affect the monomers‐to‐tetramer hLDH‐A transition. Moreover, by taking advantage of the isolation of monomeric hLDH‐A at neutral pH, we tested the effectiveness of cyclic peptides and long‐chain dicarboxylates in hampering the assembly of hLDH‐5. Interestingly, the cyclic peptide LCO15 and the long‐chain dicarboxylate crocetin were found to be very effective inhibitors of hLDH‐A: the catalytic activity of the enzyme was inhibited by 97% and 86% when these compounds were administered at 40 and 20 μM, respectively. Furthermore, the amount of lactate produced by MCF7 human cancer cells was found to decrease by 30% in the presence of 80 μM crocin (a diester of crocetin). Overall, our observations indicate that the assembly of hLDH‐A into catalytically competent hLDH‐5 can be appropriately inhibited by means of cyclic peptides and long‐chain dicarboxylates.

## 1 INTRODUCTION

Lactate dehydrogenases (LDHs) are stereospecific enzymes whose catalytic action is committed to the reversible reduction of pyruvate to L‐ or D‐lactate (Dennis & Kaplan, 1960; Everse & Kaplan, 1973; Garvie, 1980). Interestingly, the structural and functional properties of LDHs isolated from different prokaryotes can significantly diverge. Indeed, these enzymes feature dimeric or tetrameric quaternary structure, and rely on β‐NADH/β‐NAD^+^ or FADH_2_/FAD as the cofactor engaged to fulfill the redox reaction (Garvie, 1980; Steinbüchel & Schlegel, 1983). This divergence of prokaryotic LDHs extends to how their catalytic action is exerted, that is, according to Michaelis–Menten or cooperative kinetics (Arai et al., 2009; Garvie, 1980), with further complexity conferred by homotropic or heterotropic activation. Furthermore, it is important to note that oligomeric forms of bacterial LDHs containing a sub‐maximal number of subunits feature a weak, if at all, catalytic action (Jago et al., 1971; Jonas et al., 1972).

Remarkably, the structural and functional properties of prokaryotic LDHs are substantially mirrored when considering their eukaryotic counterparts (Table 1). LDHs isolated from different eukaryotes were indeed shown to be assembled as dimers or tetramers (Kaplan et al., 1956, 1960; Selander & Yang, 1970; Takenaka & Schwert, 1956), and to be assisted by β‐NADH/β‐NAD^+^ or FADH_2_/FAD as redox partners (Cristescu et al., 2008; Eventoff et al., 1977). Moreover, it was recently shown that dimeric forms of human LDH are essentially devoid of catalytic activity (Nadal‐Bufi et al., 2025). In addition, it was demonstrated that by increasing the concentration of these dimeric forms their assembly into catalytically competent tetramers can be conveniently induced (Nadal‐Bufi et al., 2025). Furthermore, it is interesting to note that tetrameric rabbit muscle LDH at 0.1 mg/mL undergoes slow dissociation (Cho & Swaisgood, 1973), and that further dilution to 0.05 mg/mL triggers a considerable release of monomers and dimers, with the tetrameric form accounting for 10% only of the total enzyme (Yamamoto & Storey, 1988). Curiously enough, the occurrence of allosteric transitions in LDHs expressed by vertebrates was early reported (Fritz, 1965), and this observation was recently interpreted as the outcome of dissociation–association events of tetrameric rabbit and human LDH (Iacovino et al., 2022; Pasti et al., 2022).

**TABLE 1:** Kinetic parameters of prokaryotic and eukaryotic lactate dehydrogenases.

Enzyme	*K*_m_ (pyruvate, mM)	*k*_cat_ (s^−1^)	Kinetics	pH of assays	References
*Lactobacillus casei*	8.2 ± 0.3 (no FBP)	94.7 ± 4.5 (no FBP)	Sigmoidal	5.5	Li et al. (2018)
*L. casei*	3.5 ± 0.1 (with FBP)	6467 ± 207 (with FBP)	Sigmoidal	5.5	Li et al. (2018)
*Geobacillus stearothermophilus*	2.8 ± 0.4 (no FBP)	118 ± 34 (no FBP)	Sigmoidal	6.5	Cai et al. (2025)
*G. stearothermophilus*	0.5 ± 0.1 (with FBP)	64 (with FBP)	Sigmoidal	6.5	Cai et al. (2025)
*Cyanobacterium aponinum*	10 (no FBP)	46 (no FBP)	Sigmoidal	7.0	Robin et al. (2023)
*C. aponinum*	0.1 (with FBP)	182 (with FBP)	Sigmoidal	7.0	Robin et al. (2023)
*Lactobacillus plantarum*	4.3 ± 0.4	2.70 ± 0.04	Hyperbolic	6.0	Feldman‐Salit et al. (2013)
*Plasmodium falciparum*	0.055 ± 0.007	96	Hyperbolic	7.5	Shoemark et al. (2007)
Bovine heart (LDH‐1)	0.041 ± 0.002	439	Hyperbolic	7.0	Yonezawa et al. (2016)
Bovine muscle (LDH‐5)	0.11 ± 0.01	524	Hyperbolic	7.0	Yonezawa et al. (2016)
Pig heart (LDH‐1)	0.12	235	Hyperbolic	8.0	Peng et al. (2014)
Pig muscle (LDH‐5)	0.6	450	Hyperbolic	7.5	Read et al. (2001)
Human heart (LDH‐1)	0.058	143	Hyperbolic	6.0	Hewitt et al. (1999)
Human muscle (LDH‐5)	0.158	260	Hyperbolic	6.0	Read et al. (2001)
Human testis (LDH‐C)	0.03	233	Hyperbolic	7.0	LeVan & Goldberg, (1991)

Abbreviation: FBP, fructose 1,6‐bisphosphate; LDH, lactate dehydrogenase.

Concerning human LDHs, different homotetramers were identified: (i) the M_4_ form (also denoted as LDH‐5, and featuring the LDH‐A subunit), mainly expressed in muscles exposed to transient hypoxic conditions, and devoted to the reduction of pyruvate to L‐lactate (Valvona et al., 2016; Woodford et al., 2020); (ii) the H_4_ form (also known as LDH‐1, and containing the LDH‐B subunit), which is predominant in aerobic tissues, where its catalytic action is mostly committed to the oxidation of L‐lactate (Read et al., 2001); (iii) the X form (LDH‐C), detected in mitochondria isolated from spermatozoa, the action of which is specific for L‐lactate (Evrev et al., 1970; LeVan & Goldberg, 1991). In addition to the enzymes responsible for the interconversion of L‐lactate and pyruvate, the presence in human mitochondria of a D‐lactate dehydrogenase was reported (De Bari et al., 2013). Nevertheless, LDH‐5 and LDH‐1 represent the isoforms of human lactate dehydrogenase to which major attention was paid so far, both structurally and functionally speaking.

Glycolysis and lactate release are of major importance for the energetic metabolism of cancer cells, whose cytosolic pH is nevertheless higher than in normal cells (White et al., 2017). Remarkably, this phenotype of malignant cells prevents the dissociation of the LDH‐5 tetramer into its LDH‐A single subunits, preserving by these means its catalytic activity (Pasti et al., 2022). An interesting link between the quaternary structure of LDH‐A and its relevance in the energetic metabolism of cancer cells has been reported (Fan et al., 2011). In particular, tyrosine 10 (Y10) of human LDH‐A (hLDH‐A) was found to be specifically phosphorylated in quite a number of human malignant cell lines (Fan et al., 2011). In addition, size exclusion chromatography (SEC) experiments demonstrated that the phosphorylation of Y10 promotes the assembly of tetrameric hLDH‐A at the expense of its monomeric and dimeric counterparts, the relative abundance of which is higher in preparations of the enzyme bearing unphosphorylated Y10 (Fan et al., 2011). Notably, these properties are phenotypically translated in the observation that the invasive potential of different types of cancer cells can be inhibited by suppressing the phosphorylation of LDH‐A at Y10 (Jin et al., 2017).

The importance of hLDH‐A in the energetic metabolism of malignant cells triggered a wide search of inhibitors of this enzyme. In particular, the active site of LDH‐A is the major target selected so far, and quite a number of competitive inhibitors mimicking pyruvate or β‐NADH were synthesized and tested (Granchi et al., 2013; Yu et al., 2001). Albeit powerful inhibitors belonging to this class were obtained (e.g., a glycolate derivative of β‐NADH: Kotlyar et al., 2010), their selectivity is rather poor. Structurally speaking, NADH‐dependent dehydrogenases do indeed share a quite high level of similarity, the occurrence of which constrains the landscape of the searchable competitive inhibitors exerting a selective action. In addition, the structural similarity of human LDH‐A and LDH‐B represents an additional restraint to the use of competitive antagonists directed against these enzymes. Therefore, a couple of allosteric hLDH inhibitors (a phthalimide and a dibenzofuran derivative, respectively) were recently synthesized, and it was shown that their action is accomplished with strong specificity, extended to the discrimination between hLDH isoforms (Friberg et al., 2020).

Notably, an additional alternative to competitive inhibitors is represented by compounds targeting protein–protein interactions. In particular, the loss of activity related to the dissociation of tetrameric LDH‐A (Hermann et al., 1981; Pasti et al., 2022) suggests the search for peptides capable to interfere with the quaternary structure of this enzyme. Peptides can indeed mimic secondary structure elements, that is, a major determinant of the mutual interactions engaged by the subunits of an oligomeric protein (Wang et al., 2021). Nevertheless, it should be mentioned that the efficiency of peptides in interfering *in vivo* with protein–protein interactions can be counteracted by their high degree of conformational freedom and by their facile degradation in host cells. However, these drawbacks can be overcome using conformationally restricted cyclic peptides and/or by including D‐amino acids in the primary structure of peptides. Accordingly, peptides represent an appealing tool to interfere with the assembly of enzymes that exclusively feature catalytic activity in their oligomeric state. When LDHs are considered, this strategy was first tested using different peptides targeting the C‐terminal region of hLDH‐A (Jafary et al., 2019), and shortly thereafter very elegant work was performed to identify peptides capable of binding human LDH‐1 with high affinity (Thabault et al., 2020). Interestingly, an additional oligomerization site of hLDH‐B was identified, and a hexadecapeptide directed against this target was observed to bind dimeric hLDH‐B with a *K*_D_ equal to 240 μM (Thabault et al., 2021). Furthermore, it was shown that appropriately designed peptides are effective in inhibiting the activity of human LDH‐A (Nadal‐Bufi et al., 2021). In particular, a heptadecapeptide (denoted cGmC9) was found to inhibit, according to a half‐maximal inhibitory concentration (IC_50_) value equal to 2.5 μM, the activity of hLDH‐5 exerted at the expense of pyruvate. Importantly enough, the cGmC9 peptide inhibits hLDH‐5 independently of substrate concentration, therefore ruling out a competitive action for this peptide. However, it should be noted that the inhibition of hLDH‐5 by the cGmC9 peptide was determined using the tetrameric enzyme exposed to pH 2.5 (in order to dissociate its subunits and induce the peptide to bind them under these conditions), and subsequently shifting the pH to 7.4 (Nadal‐Bufi et al., 2021). Hence, this procedure suffers the inconvenient of exposing the target enzyme to harsh pH conditions, under which the peptide‐enzyme association is assayed. To provide a convenient tool for testing under physiological conditions the capability of candidate peptides to inhibit hLDH‐A activity, we isolated homogeneous monomeric hLDH‐A at neutral pH (Stefan et al., 2024). In addition, we prepared quite a number of linear and cyclic peptides, and we found that both linear and cyclic peptides mimicking the C‐terminal region of hLDH‐A were effective in inhibiting this enzyme *in vitro*, with their action being selective *in cellulo* (Stefan et al., 2024). More recently, we also reported that long‐chain dicarboxylates are competent in hampering the assembly of monomeric hLDH‐A *in vitro* (Stefan et al., 2026).

It was long ago recognized by means of gel filtration experiments that the presence of 100 μM β‐NADH and 10 mM pyruvate preserves tetrameric rabbit LDH from its disassembly (Trausch, 1972). In addition, β‐NADH was found to be proficient in containing the dissociation of chicken and heart tetrameric LDH (Di Sabato & Kaplan, 1964). Furthermore, the assembly of oligomeric LDHs was analyzed using enzyme preparations subjected to the disruption of quaternary structure induced by urea or guanidinium chloride (Chattopadhyay et al., 1994; Rudolph et al., 1977; Yamamoto et al., 2017). Albeit very interesting, these former investigations do not provide direct observations of the assembly of monomeric LDHs into their corresponding oligomeric counterparts. Therefore, we considered it of interest to inspect the kinetics of the oligomerization of monomeric hLDH‐A. Accordingly, here we report on the effects triggered by the ionic strength and β‐NADH on the assembly of hLDH‐5, and on the outcome of stopped‐flow assays designed to compare the binding of the redox cofactor to monomeric and tetrameric hLDH‐A. The inhibition exerted by cyclic peptides and long‐chain dicarboxylates on the catalytic action of hLDH‐5 is also shown.

## 2 RESULTS

### 2.1 Isolation of monomeric hLDH‐A and factors promoting its assembly into hLDH‐5

**FIGURE 1:**
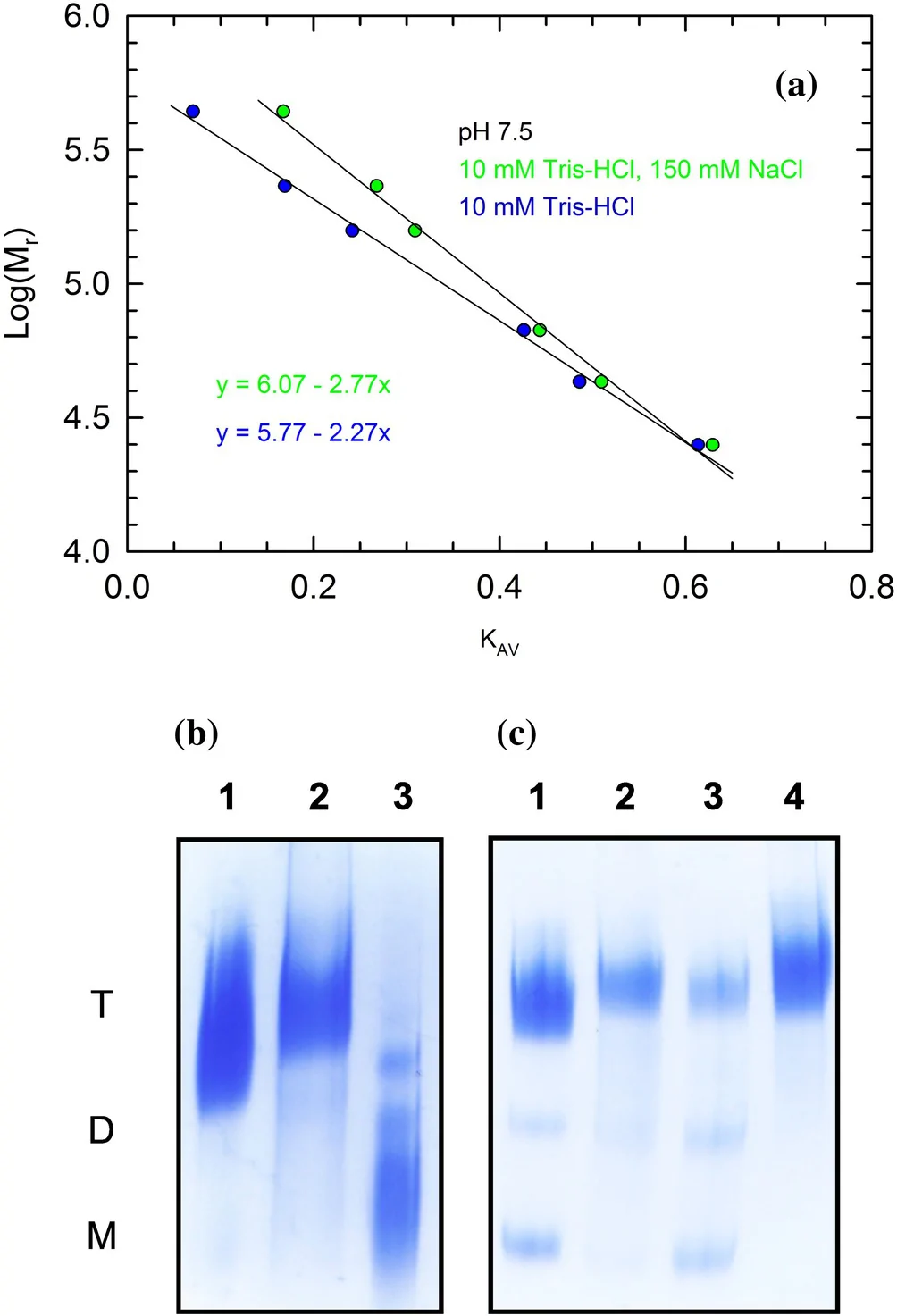
Calibration of the chromatographic column utilized to isolate monomeric human lactate dehydrogenase A (hLDH‐A) and blue‐native electrophoresis of different lactate dehydrogenases. (a) Elution of molecular mass markers from a Superdex 200 column (1.6 × 60 cm) conditioned with 10 mM Tris–HCl (pH 7.5) containing or not 150 mM NaCl (green and blue circles, respectively). The mass markers used were: ferritin, catalase, aldolase, albumin, ovalbumin, and chymotrypsinogen. (b, c) Blue‐native electrophoretic analyses performed with: tetrameric rabbit skeletal muscle LDH (rbLDH), tetrameric human muscle LDH‐A (hLDH‐5), and monomeric human muscle LDH (hLDH‐A) in 10 mM Tris–HCl (pH 7.5), loaded onto lanes 1–3, respectively (b); tetrameric rbLDH, hLDH‐5, hLDH‐A in PBS buffer, and hLDH‐A in PBS buffer supplemented with 125 μM β‐NADH loaded onto lanes 1–4, respectively (c). All the samples containing tetrameric rbLDH or hLDH‐5 were in 10 mM Tris–HCl, 150 mM NaCl, pH 7.5.

We have previously reported that monomeric hLDH‐A can be conveniently purified from *Escherichia coli* cells subjected to the overexpression of this enzyme (Stefan et al., 2024). In particular, SEC was selected as the last purification step to be performed with a Superdex 200 column conditioned with NaCl‐free 10 mM Tris–HCl, pH 7.5 (Stefan et al., 2024). Curiously enough, the elution volume of monomeric hLDH‐A did essentially correspond to that observed for chymotrypsinogen, the molecular mass of which is 30% lower compared to that expected for hLDH‐A (Stefan et al., 2024). However, mass spectrometric analyses identified the isolated monomeric hLDH‐A as the full‐length enzyme (Stefan et al., 2024). Hence, to further investigate the discrepancy between the expected and the observed molecular mass of monomeric hLDH‐A, we calibrated our Superdex 200 column using 10 mM Tris–HCl (pH 7.5), containing or not 150 mM NaCl. Our observations might appear surprising: all the molecular mass markers used to calibrate the column were eluted earlier in the absence of NaCl, with this effect being more pronounced for the subset of the three proteins whose molecular mass is higher (Figure 1a). Therefore, the delayed elution of monomeric hLDH‐A under NaCl‐free conditions represents an unexpected outcome, which we attribute to strong interactions between the enzyme and the chromatographic beads.

To assess the effect, if any, exerted by different factors on the assembly of monomeric hLDH‐A into its tetrameric form, blue‐native electrophoresis analyses were carried out using both rabbit and human LDH‐5 as appropriate controls (Figure 1b). First, and not surprisingly, we found that monomeric hLDH‐A can be easily converted into tetrameric hLDH‐5 by exchanging the storage buffer, for example, replacing 10 mM Tris–HCl with phosphate‐buffered‐saline (PBS) (Figure 1c, lane 3). This finding is in agreement with our previous SEC analyses, according to which the addition of 150 mM NaCl triggers the assembly of monomeric hLDH‐A into hLDH‐5 (Stefan et al., 2024). It should however be noted that a fraction of monomeric hLDH‐A, as well as of its dimeric form, persists in the enzyme stored in PBS buffer (Figure 1c, lane 3). Nevertheless, the addition of β‐NADH to hLDH‐A stored in PBS promoted the complete conversion of the monomeric enzyme into hLDH‐5 (Figure 1c, lane 4).

**FIGURE 2:**
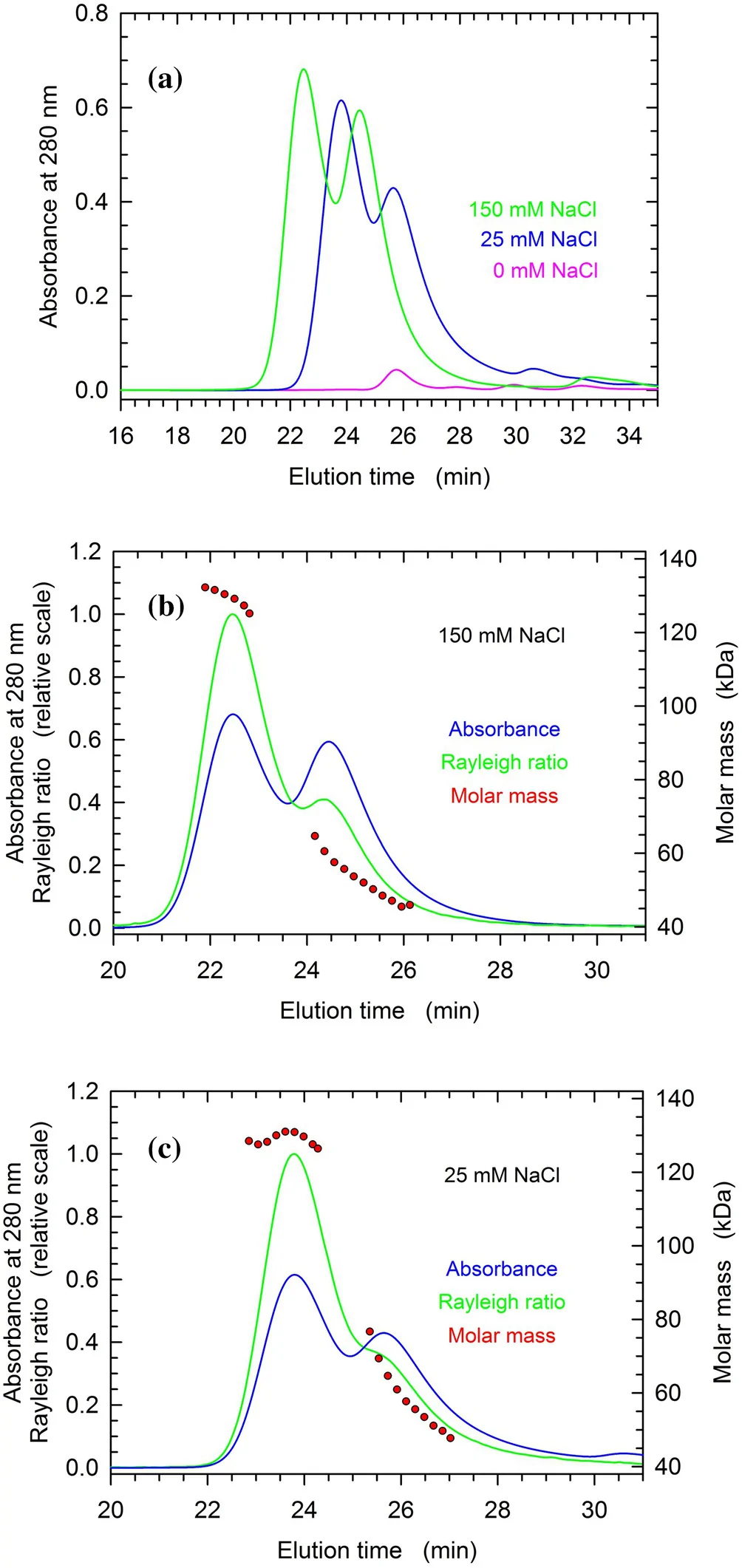
Determination of the molecular mass of human lactate dehydrogenase A (hLDH‐A) oligomers by means of size exclusion chromatography coupled to multiangle light scattering detection. (a) Chromatographic peaks detected by loading 32 μM monomeric hLDH‐A exposed for 2 h to 25 or 150 mM NaCl (blue and green line, respectively). A control sample not exposed to NaCl is also shown (magenta line). All samples were buffered with 10 mM Tris–HCl, pH 7.5. (b, c) The absorbance (blue line), Rayleigh ratio (green line), and estimated molar mass (red circles) are shown as a function of the elution time from the size exclusion column onto which was loaded monomeric hLDH‐A exposed for 2 h to 150 (b), or to 25 (c) mM NaCl.

To analyze how the presence of NaCl affects the oligomerization of monomeric hLDH‐A, we performed SEC experiments using a Superose 12 column coupled to a Multi‐Angle Light Scattering (MALS) detector. In particular, a single batch of homogeneous monomeric hLDH‐A (eluted from a Superdex 200 column conditioned with NaCl‐free 10 mM Tris–HCl, pH 7.5) was split into three aliquots, to which 0, 25, or 150 mM NaCl was added. Each aliquot was then subjected to SEC‐MALS, with the chromatographic column conditioned with the same NaCl concentration of the sample. When monomeric hLDH‐A was exposed to 150 mM NaCl, a considerable fraction of the enzyme loaded onto the column was recovered within a peak the elution time of which was 22.5 min (Figure 2a and Table 2). Moreover, a thorough inspection of this peak indicates the presence of a monodisperse protein population, featuring a molecular mass equal to 129 kDa, a value compatible with that expected for hLDH‐5, that is, 147 kDa (Figure 2b and Table 2). Interestingly, under these conditions of ionic strength the presence of an additional peak was detected (Figure 2b), and its analysis suggest that it corresponds to a non‐resolved component containing both dimeric and monomeric hLDH‐A (Figure 2b). The observations obtained in the presence of 25 mM NaCl were similar to those achieved at the higher ionic strength (Figure 2a,c), albeit the two detected peaks featured a less pronounced absorbance (Figure 2a and Table 2). Surprisingly enough, when NaCl was not added to monomeric hLDH‐A and it was omitted from the buffer (10 mM Tris–HCl) used for the chromatographic run, we detected only a tiny peak, the elution time of which was similar to that of the peak observed in the presence of 25 mM NaCl (Figure 2a).

**TABLE 2:** Features of the primary and secondary peaks detected by size exclusion chromatography‐Multi Angle Light Scattering analysis.

Sample	25 mM NaCl	150 mM NaCl		25 mM NaCl
			25 mM NaCl	125 μM β‐NADH
			125 μM β‐NADH	5 mM oxamate
Peak 1				
Elution time (min)	23.8	22.5	23.0	23.0
Absorbance at max	0.615	0.681	0.969	1.000
*M*_r_ at max (kDa)	131.0	129.0	134.0	135.5
Peak 2				
Elution time (min)	25.6	24.5	26.0	25.7
Absorbance at max	0.429	0.594	0.237	0.240
*M*_r_ at max (kDa)	67.5	58.4	55.8	58.6
Recovery (Peak 1 and Peak 2, mg)				
Determined by UV	0.17794	0.20570	0.19568	0.19896

**FIGURE 3:**
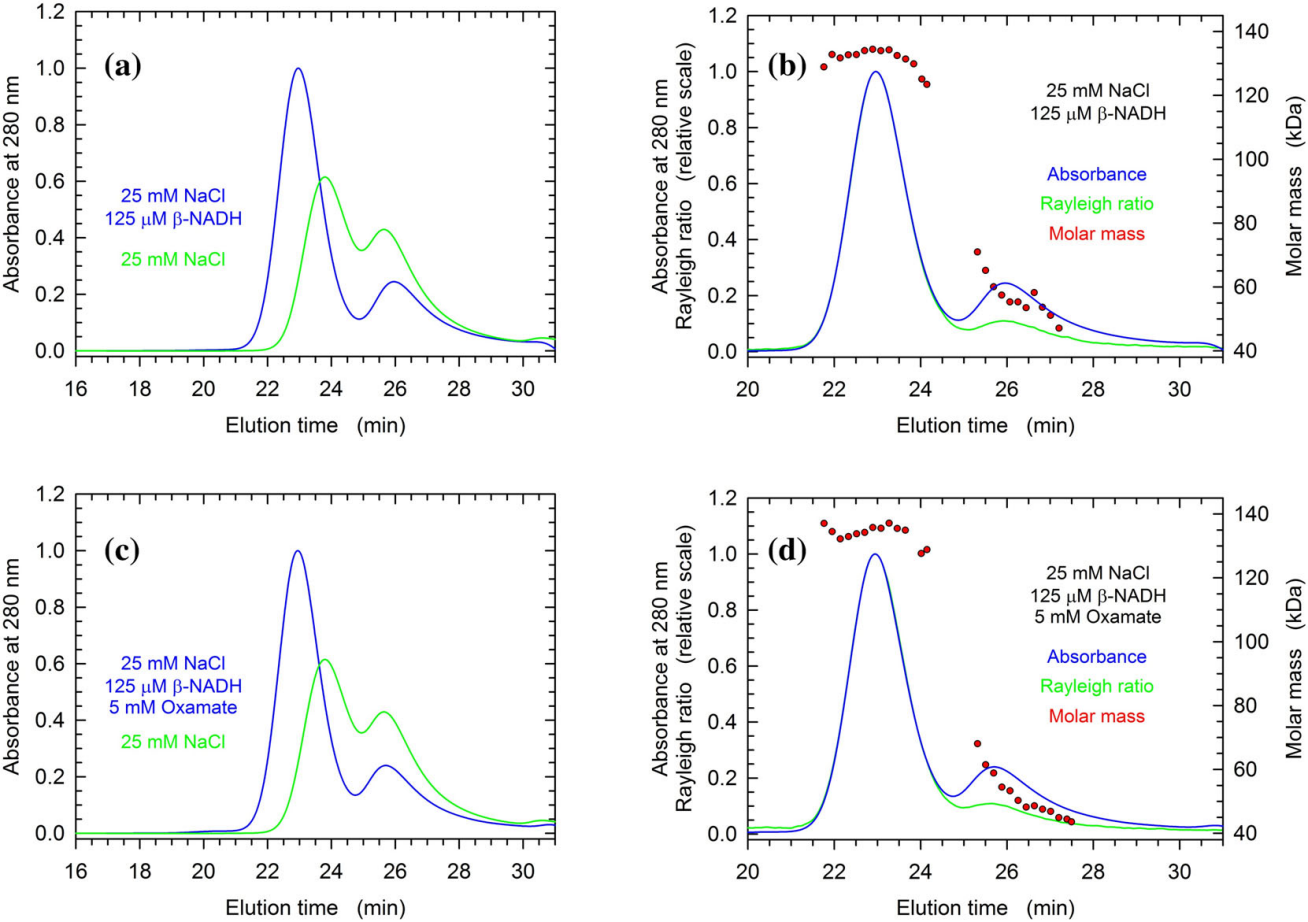
Oligomerization of monomeric human lactate dehydrogenase A (hLDH‐A) induced by β‐NADH in the absence or in the presence of oxamate. (a) Chromatographic peaks detected by loading 32 μM monomeric hLDH‐A exposed for 2 h to 25 mM NaCl, and 125 μM β‐NADH (blue line). A control sample (also shown in Figure 2) exposed to 25 mM NaCl only is also reported (green line). All samples were buffered with 10 mM Tris–HCl, pH 7.5. (b) The absorbance (blue line), Rayleigh ratio (green line), and estimated molar mass (red circles) are shown as a function of the elution time from the size exclusion column onto which was loaded monomeric hLDH‐A exposed for 2 h to 25 mM NaCl and 125 μM β‐NADH. (c) Chromatographic peaks detected by loading 32 μM monomeric hLDH‐A exposed for 2 h to 25 mM NaCl, 125 μM β‐NADH, and 5 mM oxamate (blue line). A control sample (also shown in Figure 2) exposed to 25 mM NaCl only is also reported (green line). All samples were buffered with 10 mM Tris–HCl, pH 7.5. (d). The absorbance (blue line), Rayleigh ratio (green line), and estimated molar mass (red circles) are shown as a function of the elution time from the size exclusion column onto which was loaded monomeric hLDH‐A exposed for 2 h to 25 mM NaCl, 125 μM β‐NADH, and 5 mM oxamate.

We also inspected the competence of β‐NADH and oxamate (a pyruvate‐mimicking competitive inhibitor) in promoting the assembly of hLDH‐5. As a first test, we subjected to SEC‐MALS a sample of monomeric hLDH‐A in 10 mM Tris–HCl, 25 mM NaCl (pH 7.5), and supplemented with 125 μM β‐NADH. This NaCl‐ and cofactor‐containing buffer was also used to condition our Superose 12 column and to perform the chromatographic run. Notably, the addition of β‐NADH to the enzyme solution was found to effectively promote the conversion of hLDH‐A into hLDH‐5 (Figure 3a,b). The presence of the redox cofactor did indeed significantly increase the fraction of tetrameric hLDH‐A over that observed in the enzyme solution to which only 25 mM NaCl was provided (Figure 3a and Table 2). By means of a further test, we observed that the addition of both 125 μM β‐NADH and 5 mM oxamate to monomeric hLDH‐A in 10 mM Tris–HCl, 25 mM NaCl (pH 7.5) induced the assembly of hLDH‐5 to an extent similar to that observed in the presence of the cofactor only (Figure 3c,d; Table 2).

It is important to mention that the overall hLDH‐A recovery (monomer, dimer, and tetramer) from the column was essentially consistent among the different samples. Indeed, the divergence detected for the recovery values did not exceed 13.5%, as observed between the experiments performed in the presence of 25 and 150 mM NaCl (Table 2).

### 2.2 The binding of β‐NADH triggers the assembly of hLDH‐5

Taking into account previous reports indicating that β‐NADH and pyruvate (or oxamate) induce the generation of tetrameric LDH‐A (Stefan et al., 2024; Trausch, 1972), we considered it of interest to investigate the kinetics of the action exerted by the redox cofactor on the assembly of hLDH‐5. To this aim, we performed stopped‐flow assays by exposing to β‐NADH monomeric and tetrameric hLDH‐A. It should be mentioned here that the binding of the redox cofactor translates into the quenching of the fluorescence emitted by the tryptophanes of lactate dehydrogenase, according to an intra‐molecular Förster Resonance Energy Transfer (FRET) mechanism (Szabó et al., 2022). Moreover, the conformational rearrangements triggered by the binding of β‐NADH might also imply a decrease in the tryptophanes' fluorescence intensity (Reddish et al., 2017). As a first test, we analyzed how the binding of β‐NADH affects the fluorescence of hLDH‐A tryptophanes as a function of the excitation wavelength. Not surprisingly, the amplitude of the observed excitation spectrum features a minimum at 280–290 nm, whereas the values for the binding rate constant were determined as essentially invariant with respect to the excitation wavelength (Figure S1). Taking into account these observations, we selected 280 nm, which represents an isosbestic point for β‐NADH/β‐NAD^+^ (Figure S2), as an excitation wavelength suitable to analyze the fluorescence of hLDH‐A tryptophanes.

**FIGURE 4:**
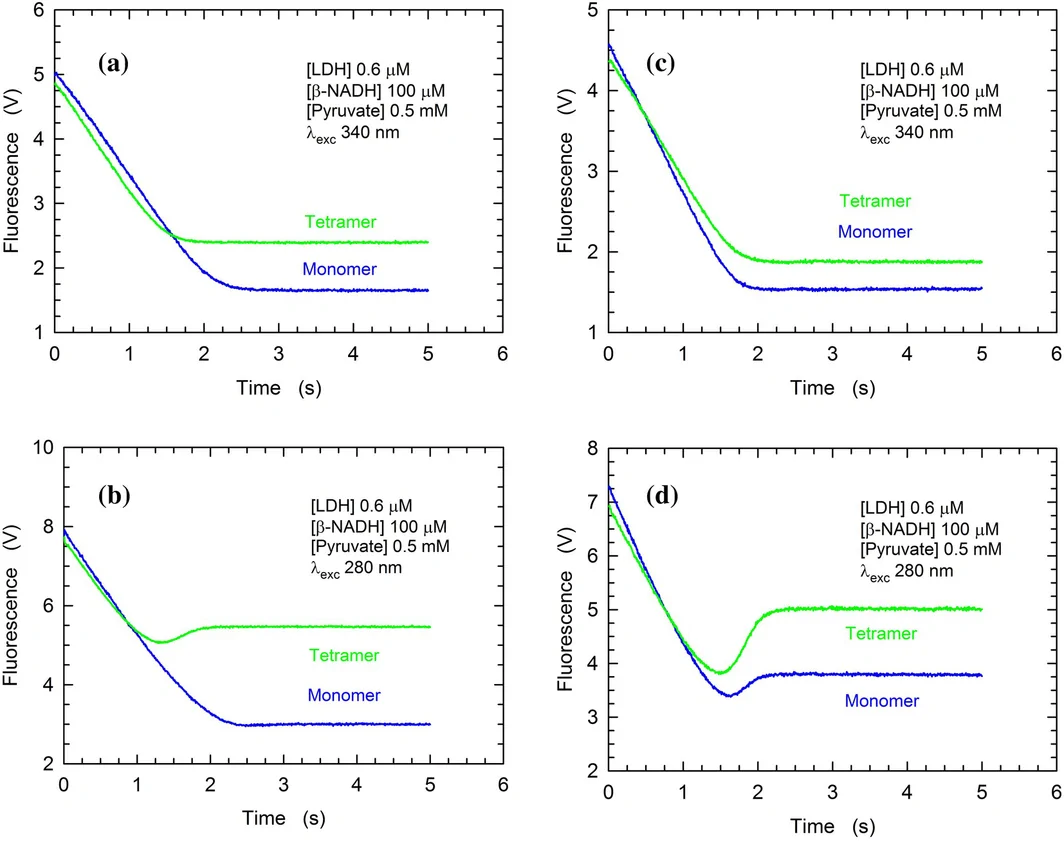
Stopped‐flow assays of pyruvate reduction catalyzed by monomeric and tetrameric human lactate dehydrogenase A (hLDH‐A). (a) Kinetics of the β‐NADH oxidation linked to the reduction of pyruvate to L‐lactate catalyzed by tetrameric (green line) and monomeric (blue line) human muscle lactate dehydrogenase. Samples were excited with light of 340 nm. The enzyme syringe contained 1.2 μM enzyme, and the substrate syringe was filled with 200 μM β‐NADH and 1 mM pyruvate. Both solutions were buffered with 10 mM Tris–HCl, pH 7.5. (b) Time‐course of the fluorescence changes detected exciting samples with light of 280 nm. The enzyme syringe contained 1.2 μM enzyme (tetrameric or monomeric, green and blue line, respectively), and the substrate syringe was filled with 200 μM β‐NADH and 1 mM pyruvate. Both solutions were buffered with 10 mM Tris–HCl, pH 7.5. (c) Kinetics of the β‐NADH oxidation linked to the reduction of pyruvate to L‐lactate catalyzed by tetrameric (green line) and monomeric (blue line) human muscle lactate dehydrogenase. Samples were excited with light of 340 nm. The enzyme syringe contained 1.2 μM enzyme and 200 μM β‐NADH, and the substrate syringe was filled with 1 mM pyruvate. Both solutions were buffered with 10 mM Tris–HCl, pH 7.5. (d) Time‐course of the fluorescence changes detected exciting samples with light of 280 nm. The enzyme syringe contained 1.2 μM enzyme (tetrameric or monomeric, green and blue line, respectively) and 200 μM β‐NADH, and the substrate syringe was filled with 1 mM pyruvate. Both solutions were buffered with 10 mM Tris–HCl, pH 7.5.

As previously mentioned, the catalytic action of LDHs is known to strictly depend on their quaternary structure. The breakdown of tetrameric LDH‐A and LDH‐B leads to a loss of enzymatic activity, which can be restored by incubating the separated subunits under conditions that promote their reassembly (Bartholmes et al., 1973; Hermann et al., 1981; Pasti et al., 2022). Hence, we compared the catalytic action exerted by tetrameric hLDH‐A with the activity of its monomeric counterpart exposed to β‐NADH and therefore assembled into hLDH‐5 (see Figure 3). In particular, two distinct stopped‐flow assays were carried out, by filling: (i) the enzyme syringe with hLDH‐A only and the substrate syringe with β‐NADH and pyruvate; (ii) the enzyme syringe with hLDH‐A and β‐NADH, and the substrate syringe with pyruvate only. Furthermore, both these assays were performed in duplicate, that is, exciting the mixed solutions with light of 340 nm (to determine the oxidation of β‐NADH) or 280 nm (to detect the fluorescence of enzyme tryptophanes). When the solutions containing hLDH‐A only were mixed with β‐NADH and pyruvate, and the samples were excited with light of 340 nm, the oxidation of 100 μM β‐NADH was completed by the tetrameric enzyme in less than 2 s (Figure 4a), whereas the reaction observed in the presence of monomeric hLDH‐A required about 2.5 s to reach equilibrium (Figure 4a). Interestingly, this difference was also detected when the samples were exposed to light of 280 nm (Figure 4b) which is known to excite both tryptophan and the redox cofactor, whose emission was shown to increase when associated with beef heart LDH (Velick, 1958; see Figures S3 and S4 for β‐NADH bound to hLDH‐5). Taking into account these features of β‐NADH, the kinetics we observed can be conveniently interpreted. When the tetrameric enzyme was considered, an initial decrease of fluorescence was detected, after which a rapid increase led, within 2 s after mixing, to an apparent steady‐state (Figure 4b). Conversely, when monomeric hLDH‐A was used, no fluorescence increase was observed, and the steady‐state was attained in about 2.5 s (Figure 4b). In both assays, the fluorescence decrease is due to the oxidation of β‐NADH and to the quenching of tryptophanes' emission, whose fluorescence recovers upon the consumption of the redox cofactor (Figure 4b). Moreover, the absence of an emission increase phase in the kinetics observed in the presence of monomeric hLDH‐A suggests that the redox reaction is not completed. Accordingly, we found that a significant, albeit small, late decrease of β‐NADH concentration occurs in the assay mixture containing the monomeric enzyme (Figure S5).

Remarkably, when the enzyme syringe of the stopped‐flow did also contain β‐NADH, the oxidation of the redox cofactor was completed in about 2 s, independently of the enzyme form used in the assay (Figure 4c). The similarity between the time‐course of the reactions observed in the presence of monomeric and tetrameric hLDH‐A was also detected when the samples were excited with light of 280 nm (Figure 4d). Indeed, the major difference between the kinetics of the reactions catalyzed by hLDH‐5 and monomeric hLDH‐A resides in the amplitude of both the initial fluorescence decrease (irrespective of the excitation wavelength) and the late increase of tryptophanes' fluorescence (Figure 4c,d).

### 2.3 Kinetics of β‐NADH binding by monomeric and tetrameric hLDH‐A

**FIGURE 5:**
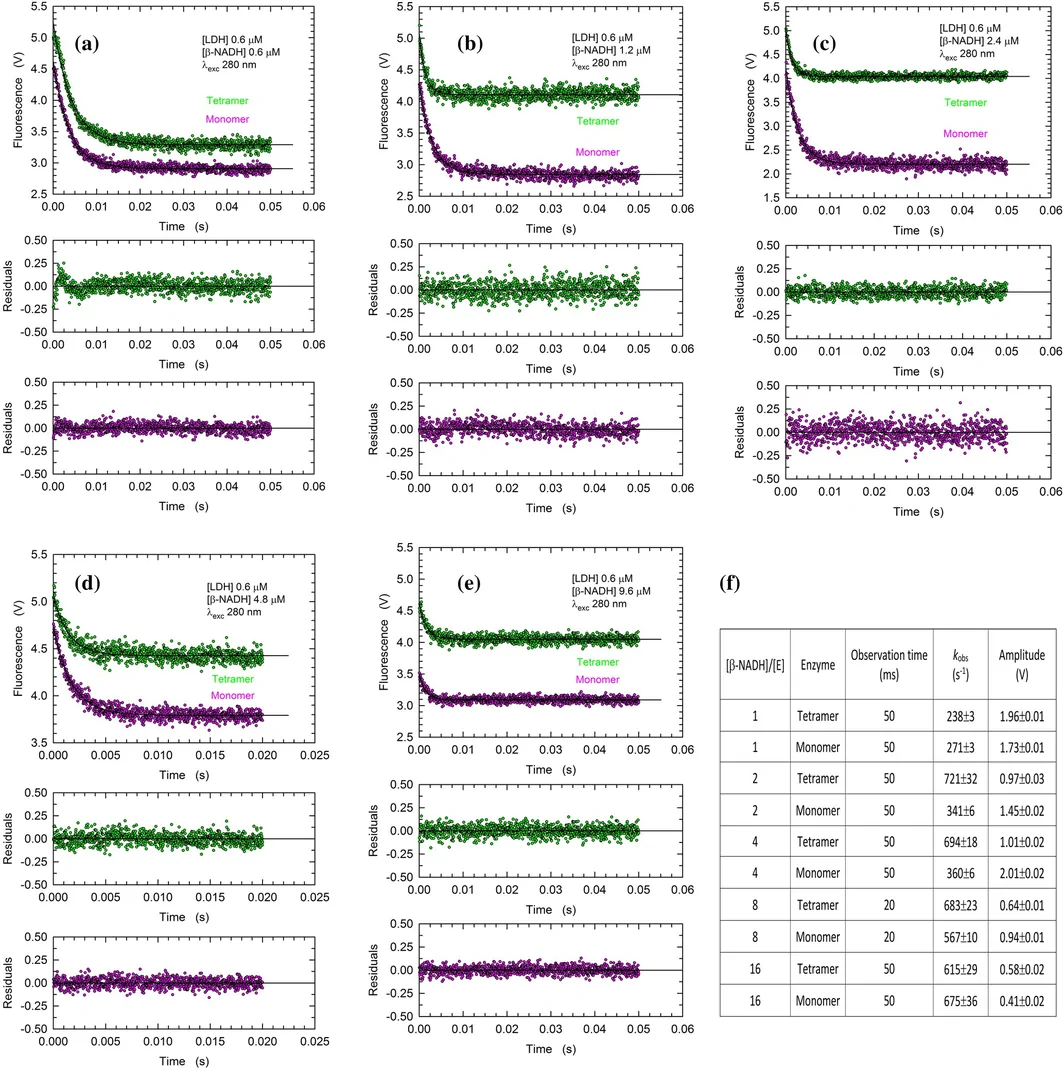
Kinetics, rate constants, and amplitudes of the fluorescence changes of human lactate dehydrogenase A (hLDH‐A) tryptophanes induced by mixing 1.2 μM enzyme with β‐NADH. (a–e) Kinetics of the fluorescence quenching, triggered by β‐NADH binding, of tetrameric (green circles) and monomeric (magenta circles) hLDH‐A tryptophanes. The enzyme syringe was filled with 1.2 μM enzyme, and the substrate syringe contained 1.2, 2.4, 4.8, 9.6, or 19.2 μM β‐NADH (a–e panels, respectively). The black lines represent the best fit of a single exponential equation to the experimental observations. (f) Rate constants and amplitudes determined by the best fits shown in panels (a–e).

**FIGURE 6:**
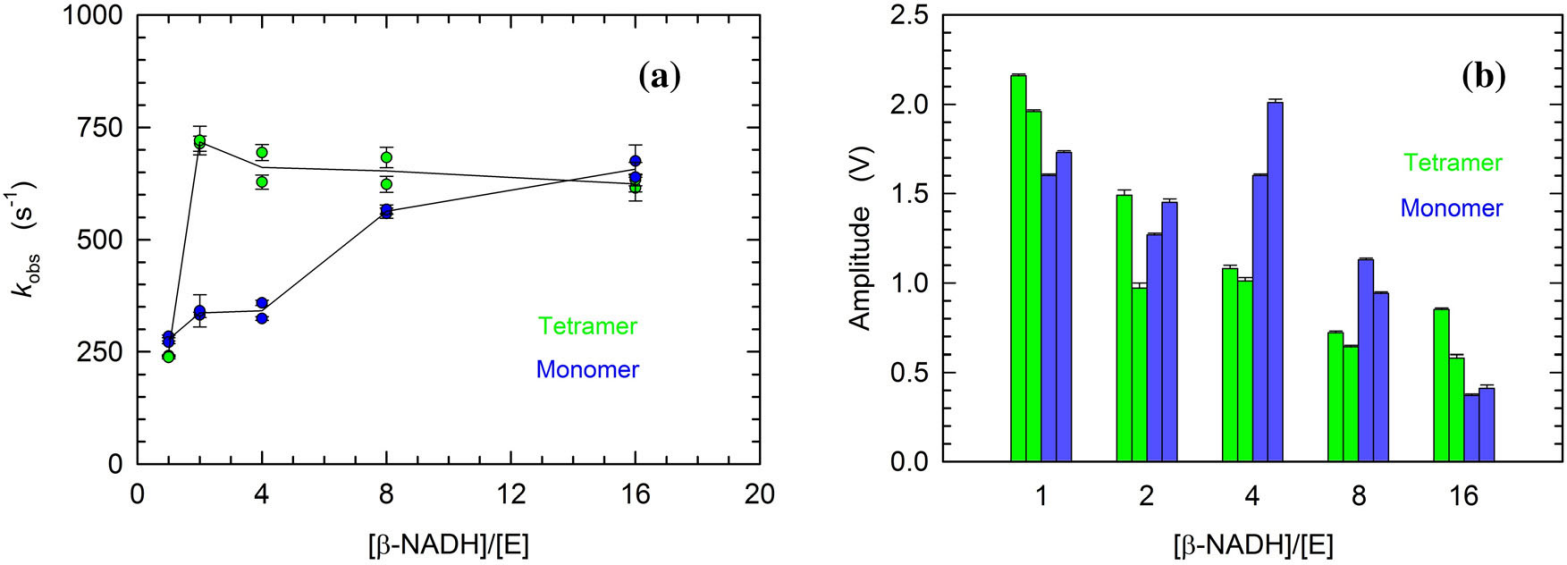
Rate constants and amplitudes related to the fluorescence changes of human lactate dehydrogenase A (hLDH‐A) tryptophanes induced by β‐NADH binding. (a, b) Rate constants (a) and amplitudes (b) determined by fitting a single exponential equation to the observed fluorescence changes of tetrameric (green circles and bars) and monomeric (blue circles and bars) hLDH‐A tryptophanes induced by β‐NADH binding under different conditions of β‐NADH/enzyme molar ratio, and shown in Figures 5, S6, and S7. The error bars represent the standard deviation associated with each fitting.

The stopped‐flow assays previously shown (Figure 4) indicate that the exposure to β‐NADH prompts monomeric hLDH‐A to exert a catalytic action which is comparable to that featured by hLDH‐5. However, under the conditions of these assays the redox cofactor is continuously oxidized (Figure 4), and therefore no information can be obtained about the β‐NADH/hLDH‐A molar ratio which is suitable to convert the monomeric enzyme into its tetrameric counterpart. To investigate this point, we analyzed the kinetics of β‐NADH binding by means of stopped‐flow experiments, performed as a function of the redox cofactor concentration in the presence of monomeric or tetrameric hLDH‐A. Moreover, each assay was carried out twice, sometimes using two different time sweeps. When β‐NADH and hLDH‐5 were mixed at equimolar concentration, the association reaction was found to be rather slow, featuring a *k*_obs_ equal to 238 ± 3 (Figure 5a,f) and 241 ± 2 s^−1^ (Figures S6A and S7). However, by doubling the concentration of β‐NADH we determined quite larger values for the rate constant of the binding reaction, that is, 721 ± 32 (Figure 5b,f) and 714 ± 17 s^−1^ (Figures S6B and S7). Interestingly enough, further doublings of the β‐NADH concentration (up to a cofactor/enzyme molar ratio equal to 16) did not trigger any additional increase of the reaction velocity (Figures 5c–f, S6C–E, and S7). Remarkably, the assays performed with monomeric hLDH‐A indicated a different dependence of the observed rate constant on β‐NADH concentration. Indeed, only when the concentration of the redox cofactor was 16 times in excess respect the concentration of hLDH‐A the observed rate constant attained a maximum value, comparable to that determined for hLDH‐5 (Figures 5, 6A, S6, and S7). When the amplitudes of the binding reactions are considered, a significant divergence between the observations obtained with hLDH‐5 and those achieved in the presence of monomeric hLDH‐A clearly appears. In particular, the maximum value for the amplitude of the binding reaction performed by hLDH‐5 and by monomeric hLDH‐A was found when the β‐NADH/enzyme molar ratio was equal to 1 and 4, respectively (Figure 6b).

### 2.4 Peptides and dicarboxylates preclude the genesis of tetrameric hLDH‐5

**FIGURE 7:**
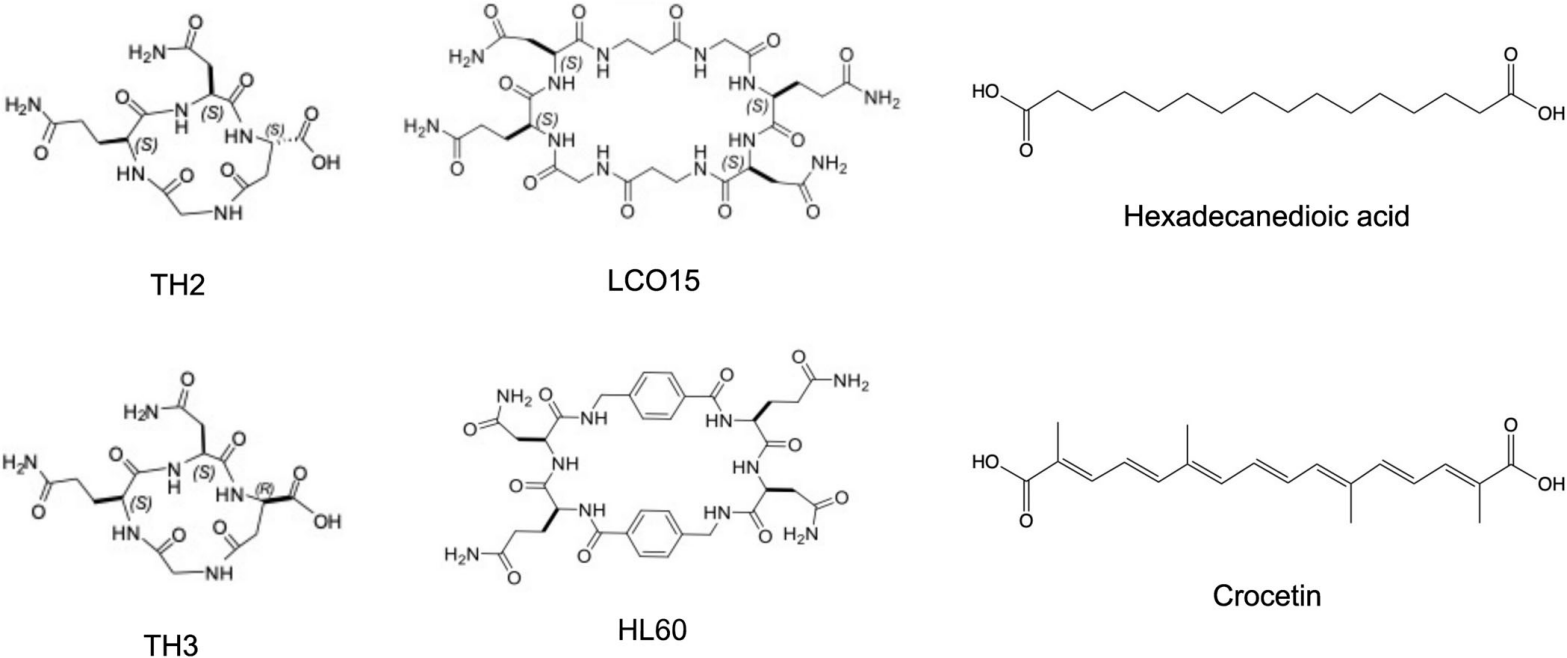
Cyclic peptides and long‐chain dicarboxylates assayed as candidate inhibitors of human lactate dehydrogenase A.

**FIGURE 8:**
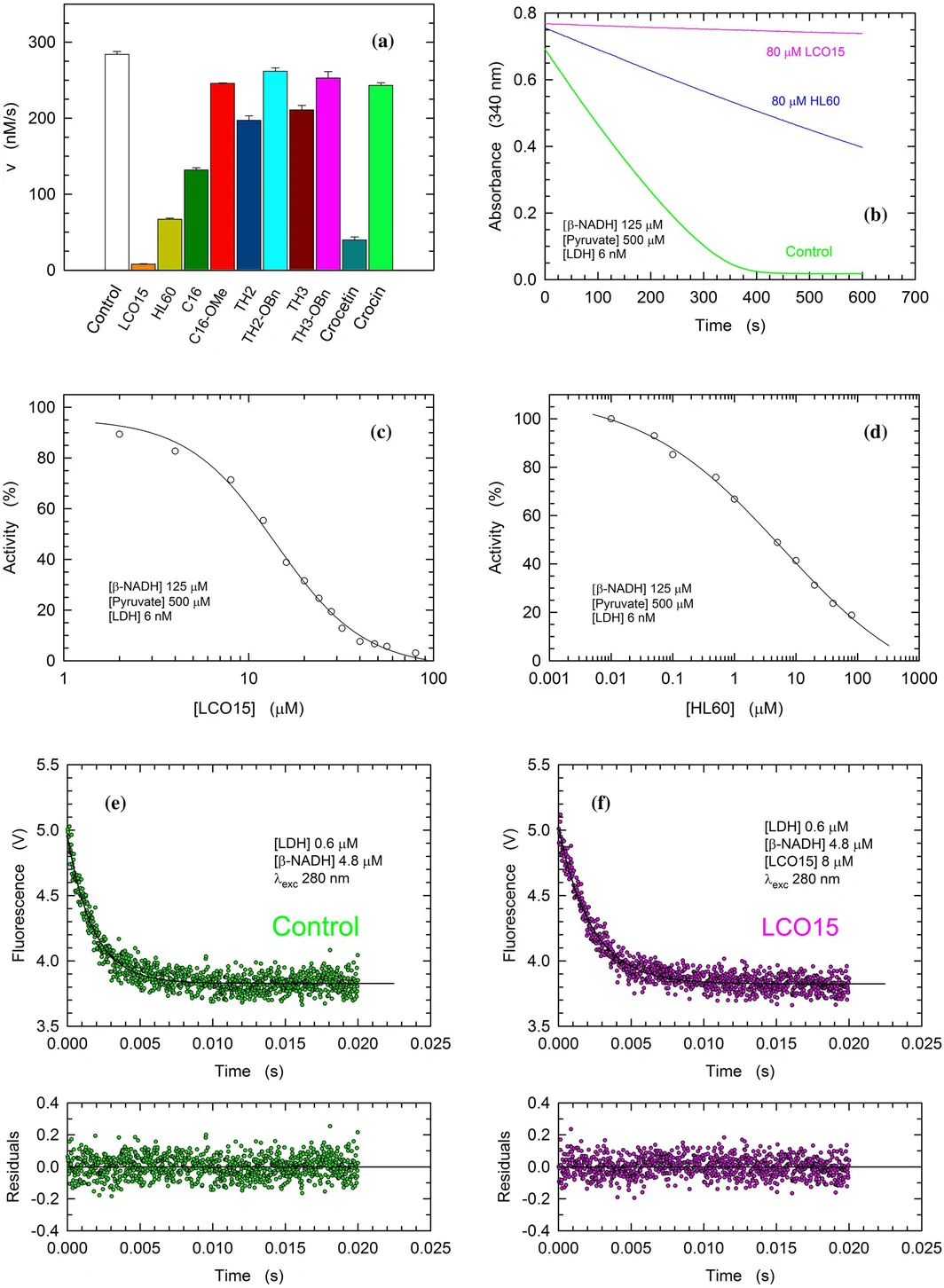
Inhibition exerted by peptides and dicarboxylates on the catalytic activity and on the binding of β‐NADH, featured by monomeric human muscle lactate dehydrogenase. (a) Competence of the indicated compounds in inhibiting the activity induced in monomeric human lactate dehydrogenase A (hLDH‐A) (6 nM) when exposed to 125 μM β‐NADH and 500 μM pyruvate (in 10 mM Tris–HCl, pH 7.5). The candidate inhibitors were added to reaction mixtures at 80 μM, with the exception of the LCO15 peptide, crocetin, and crocin that were tested at 40, 20, and 20 μM, respectively. The error bars represent standard deviation (*n* = 3). (b) Kinetics of β‐NADH oxidation observed in the presence of 6 nM monomeric hLDH‐A, 125 μM redox cofactor, and 500 μM pyruvate (green line). The time‐course of the same reaction detected using assay mixtures supplemented with 80 μM of the LCO15 or the HL60 peptide (magenta and blue line, respectively) is also shown. (c, d) Catalytic activity of human monomeric LDH‐A as a function of the concentration of the LCO15 (c) and HL60 (d) cyclic peptide. Other reaction conditions as in panels (a) and (b). (e, f) Kinetics of the fluorescence changes induced in monomeric hLDH‐A tryptophanes by the binding of β‐NADH, in the absence (e) or in the presence (f) of the LCO15 cyclic peptide. The stopped‐flow assays were performed by mixing 1.2 μM enzyme with 9.6 μM β‐NADH (and 16 μM peptide when present). All solutions were buffered with 10 mM Tris–HCl, pH 7.5. The continuous lines represent the best fit of a single exponential equation to the experimental observations.

As already noted, we recently isolated monomeric hLDH‐A, and this prompted us to test quite a number of peptides as candidate inhibitors directed against the assembly of hLDH‐5 (Stefan et al., 2024). In particular, two cyclic peptides, denoted TH2 and TH3 (Figure 7), were found competent in lowering by about 30% the catalytic activity of monomeric hLDH‐A exposed to 125 μM β‐NADH and 500 μM pyruvate (Stefan et al., 2024). Moreover, we observed that the presence in these peptides of a free carboxylate is essential for their inhibitory action. Indeed, their cognate benzyl esters did not feature any significant antagonism against monomeric hLDH‐A (Stefan et al., 2024). Subsequently, we also observed that long‐chain dicarboxylates such as hexadecanedioic acid (Figure 7) exert a strong inhibition of the conversion of hLDH‐A monomers into hLDH‐5 (Stefan et al., 2026). Taking into account our previous findings, we designed, synthesized, and assayed a couple of cyclic peptides (Figure 7), denoted as LCO15 and HL60, respectively featuring the primary structure GQN‐(β)Ala‐GQN‐(β)Ala and QN‐Aba‐QN‐Aba (Aba: 4‐(aminomethyl)benzoic acid). In particular, the GQN stretch of the LCO15 peptide is identical to the residues 296–298 of hLDH‐A, and therefore this triad of amino acids is predicted to interact with the N‐terminal region of the target (Stefan et al., 2024). Remarkably, the presence of LCO15 in assay mixtures was responsible for an almost complete inhibition of the catalytic activity induced in monomeric hLDH‐A by 125 μM β‐NADH and 500 μM pyruvate (Figure 8a,b). Under these assay conditions, the HL60 peptide was also identified as an effective inhibitor, being able to decrease by 76% the activity of the target (Figure 8a,b).

Furthermore, and in agreement with the data we previously reported (Stefan et al., 2024), we determined that the presence of a free carboxylate in the peptides TH2 and TH3 is essential for their inhibitory action. Indeed, the benzyl esters of TH2 and TH3 (respectively designated as TH10 and TH11) did show a modest, if at all, antagonist activity against hLDH‐A (Figure 8a). Accordingly, we considered of interest to synthesize the methyl ester of hexadecanedioc acid (C16), which we have recently shown as being competent in antagonizing monomeric hLDH‐A (Stefan et al., 2024). Interestingly, the C16 methyl ester provided a weak inhibition of the target, the activity of which was reduced by 13% only by the addition of 80 μM of this compound to reaction mixtures (Figure 8a). In addition, and in line with our previous observations (Stefan et al., 2026), a comparison between crocetin (an unsaturated C16 dicarboxylate, Figure 7) and crocin (the digentiobiose ester of crocetin) indicates that the antagonism of long‐chain dicarboxylates against hLDH‐A is strictly dependent on free carboxylic groups (Figure 8a). However, it is important to note that the carboxylate group of peptides TH2 and TH3 interacts with the residue K13 of hLDH‐A, whereas the C16 carboxylates are directed against the amino acids H271 and R269 of the monomeric enzyme (Stefan et al., 2024, 2026).

Among the LDH‐A inhibitors reported here crocetin represents the most effective dicarboxylate, and it should be noted that we determined its IC_50_ value as equal to 436 ± 36 nM (Stefan et al., 2026). In addition to crocetin, the peptides LCO15 and HL60 do also feature quite strong inhibitory action against monomeric hLDH‐A (Figure 8a). Therefore, we estimated the IC_50_ of these two cyclic peptides, obtaining values equal to 14.0 ± 0.7 and 6 ± 3 μM for LCO15 and HL60, respectively (Figure 8c,d). These values are of the same order of magnitude when compared to the IC_50_ reported for the cGmC9 heptadecapeptide, which is equal to 2.5 μM (Nadal‐Bufi et al., 2021). Nevertheless, it should be mentioned that hLDH‐A inhibitors featuring much lower IC_50_ were reported, for example, the small molecule GNE‐140, in the presence of which the catalytic action of hLDH‐A was negatively affected according to an IC_50_ equal to 3 nM (Boudreau et al., 2016).

As previously mentioned, the binding of β‐NADH to monomeric hLDH‐A triggers the assembly of four monomers into hLDH‐5. Taking account of this, to inspect the mechanism of action of the LCO15 octapeptide, we performed stopped‐flow assays to determine the kinetics of β‐NADH association to monomeric hLDH‐A, in the absence or in the presence of LCO15. When 0.6 μM enzyme was mixed with 4.8 μM β‐NADH, we detected, as expected, a rather fast kinetics, with a *k*_obs_ estimated as equal to 559 ± 11 s^−1^, and an amplitude of 1.13 ± 0.02 V (Figure 8e). Remarkably, the *k*_obs_ was found to decrease to 438 ± 7 s^−1^ when monomeric hLDH‐A was mixed with β‐NADH in the presence of 8 μM LCO15. Importantly enough, under these conditions the amplitude of the binding reaction was determined as equal to 1.19 ± 0.01 V (Figure 8f). This indicates that the extent of β‐NADH binding is not affected by the presence of the octapeptide, the action of which is limited to decrease by 20% the association rate of the redox cofactor to hLDH‐A. It is worth mentioning that the inhibition of LDH‐5 in malignant cells translates into an enhancement of the NADH/NAD^+^ ratio *in cellulo* (Erdem et al., 2025), and that this phenotype represses tumor progression (Le et al., 2010). Quantitatively speaking, the inhibition of lactate production by 30% was shown to increase the NADH/NAD^+^ ratio up to one order of magnitude (Erdem et al., 2025). It should also be mentioned that LDH was found to outperform malate dehydrogenase in β‐NADH oxidation in different malignant cell lines (Wang et al., 2022). Accordingly, we suggest that the inhibition exerted by the LCO15 octapeptide on the rate of β‐NADH binding to LDH‐A (Figure 8e,f) and on the subsequent pyruvate reduction (Figure 8a–c) could suppress the proliferation of cancer cells by limiting the oxidation of β‐NADH and increasing the NADH/NAD^+^ ratio.

### 2.5 Inhibition of lactate production by cancer cells

**FIGURE 9:**
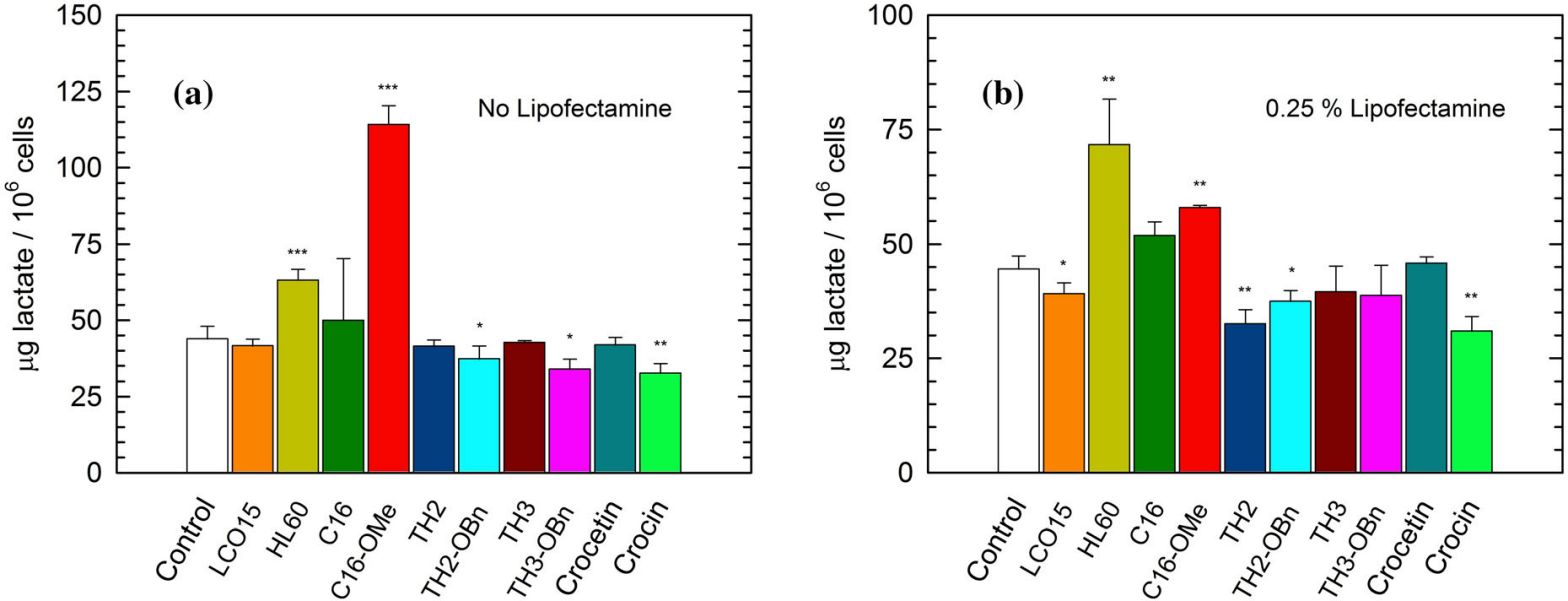
Effect of cyclic peptides and long‐chain dicarboxylates on the secretion of lactate by human cancer cells. The amount of lactate secreted by MCF7 cells in the absence (white bars) or in the presence of different cyclic peptides or long‐chain dicarboxylates is reported. All compounds were tested at 80 μM, with the exception of the cyclic LCO15 peptide, which was assayed at 40 μM. The assays were performed in the absence (a) or in the presence (b) of lipofectamine. Error bars represent standard deviation (*n* = 3). The experimental observations were compared by the Student's *t*‐test. The ***, **, and * symbols represent a *p* value lower than 0.001, 0.01, and 0.05, respectively.

The competence of peptides and dicarboxylates in repressing the activity of hLDH‐A *in cellulo* was tested using the MCF7 human breast cancer cell line, which we have previously shown to exclusively express hLDH‐A (Stefan et al., 2024). In particular, cultured MCF7 cells were exposed to 80 μM of each candidate inhibitor, in the absence or in the presence of lipofectamine. It should also be mentioned that, to limit the final dimethyl sulfoxide (DMSO) concentration at 1% (v/v), the LCO15 peptide was tested at 40 μM. When the effect, if any, induced by each compound was assayed in the absence of lipofectamine, only the benzyl ester of the TH2 and TH3 peptides, along with crocin, was found to feature a significant antagonistic action against hLDH‐A (Figure 9a). Rather surprisingly, under these conditions the peptide HL60 and the methyl ester of hexadecanedioic acid (C16‐OMe) induced an increase of lactate production, with a prominent action exerted by the latter, whose addition enhanced more than twofold the amount of lactate secreted by the cells (Figure 9a).

The presence of lipofectamine did significantly affect the action of a few candidate inhibitors, among which the C16 methyl ester featured the highest responsivity to the cationic lipid (Figure 9b), whose addition halved the increase of lactate production elicited by this compound (cf. Figure 9a,b). Moreover, the highly variable impact induced by hexadecanedioic acid on the synthesis of lactate was translated into a less fluctuating outcome by the addition of lipofectamine, whose presence generated an opposite response by the peptide HL60 (cf. Figure 9a,b). It is also interesting to note that the decrease of lactate synthesis induced by the LCO15 and TH2 peptides is lipofectamine‐dependent, whereas the antagonism against hLDH‐A featured by the TH2 benzyl ester and by crocin does not rely on the cationic lipid (cf. Figure 9a,b). Furthermore, the inhibition observed in the presence of crocin is consistent, being equal to 25 and 31% in the absence and in the presence of lipofectamine, respectively (Figure 9a,b). Accordingly, crocin represents an appealing inhibitor of hLDH‐A *in cellulo*, and we would like to suggest that its action mirrors the antagonism featured by crocetin *in vitro*.

## 3 DISCUSSION

**FIGURE 10:**
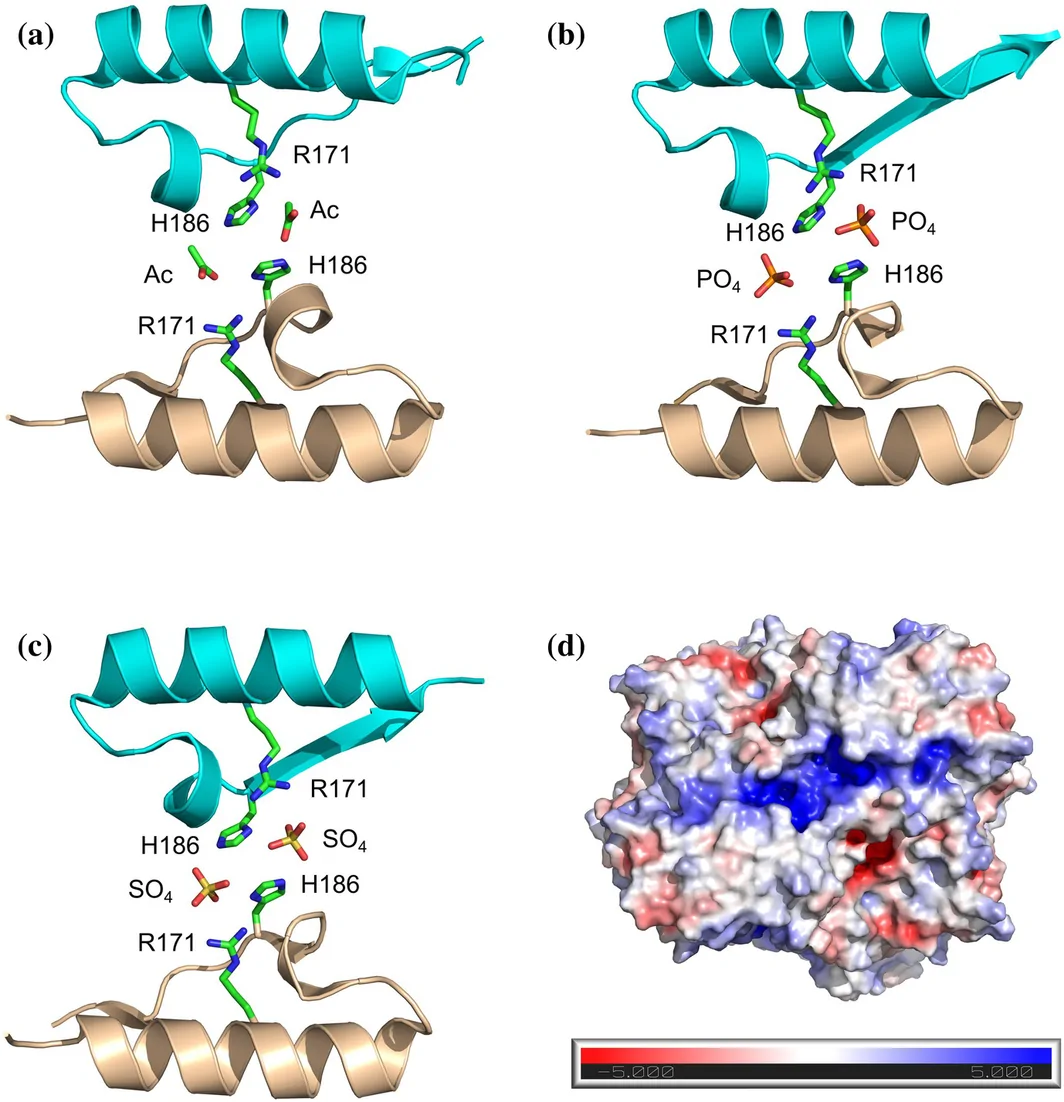
Structural features of human muscle lactate dehydrogenase. (a–c) Detail of the structural region at the interface between chains A and C (represented in cyan and golden brown, respectively) of human lactate dehydrogenase A (hLDH‐A) and containing acetate (a, Protein Data Bank (PDB)  file 1i10), phosphate (b, PDB file 6Q0D), and sulfate (c, PDB file 5iXS) ions. The coordinates of the amino acids are numbered by considering at site 1 the methionine corresponding to the start codon. (d) Surface electrostatic potential of human muscle lactate dehydrogenase (PDB file 5iXS), determined with the adaptive Poisson‐Boltzmann solver plugin of PyMol. The dark blue region corresponds to the interface between the dimers containing subunits A‐B and C‐D (located at top and bottom, respectively).

For quite a number of years, conspicuous work was committed to show that different types of dehydrogenases undergo the dissociation of their subunits when exposed to acidic conditions (Briganti et al., 1989; Chilson et al., 1965; Iacovino et al., 2022; Pasti et al., 2022; Zettlmeissl et al., 1981). Intriguingly enough, the reversible nature of this dissociation suggested its use for testing candidate peptides designed to interfere with the assembly of tetrameric human lactate dehydrogenase (Nadal‐Bufi et al., 2021). To further inspect an alternative to this strategy, we report here on our previous finding that tetrameric hLDH‐A can be induced to dissociate, at pH 7.5, by its transfer into 10 mM buffers devoid of NaCl (Stefan et al., 2024). In particular, we show by means of blue‐native electrophoresis and SEC‐MALS experiments that monomeric hLDH‐A can be converted into hLDH‐5 by the addition of NaCl to the enzyme solution (Figures 1c and 2a). To interpret this observation, we propose that chloride is the effector triggering the association of hLDH‐5 at the expense of monomeric hLDH‐A. The assembly of the tetrameric enzyme has indeed to face the electrostatic repulsion occurring between residues R171 and H186 located at adjacent subunits (Figure 10), and the presence of chloride ions bridging arginines and histidines might efficiently counteract this effect. It should also be noted that this action we propose for chloride is similar to that observed for sulfate, acetate, and phosphate (Figure 10a–c) in quaternary structures of hLDH‐5 (Purkey et al., 2016; Rai et al., 2020; Read et al., 2001). Moreover, the binding of chloride ions could help to prevent the repulsion linked to the strongly positive electrostatic potential residing along the surface of tetrameric hLDH‐A interlocking the dimers composed of subunits A–B and C–D, respectively (Figure 10d).

The competence of β‐NADH in promoting the oligomerization of LDHs was long ago recognized for bovine and chicken LDH‐1 (Chilson et al., 1965), and it was afterwards observed with human and rabbit LDH‐5 (Stefan et al., 2024; Trausch, 1972) and with human LDH‐1 (Nadal‐Bufi et al., 2025). In agreement with these observations, we found that the addition of β‐NADH to a solution of monomeric hLDH‐A in PBS buffer induces a complete conversion of four enzyme subunits into hLDH‐5 (Figure 1c). Overall, the observations obtained with blue‐native electrophoresis indicate that monomeric hLDH‐A has to be stored in a low molarity buffer (e.g., 10 mM Tris–HCl) devoid of NaCl and β‐NADH. Notably, both blue‐native electrophoresis and SEC‐MALS experiments indicated that the addition of NaCl to monomeric hLDH‐A does not induce a quantitative assembly of hLDH‐5 (Figures 1c and 2a). Nevertheless, we were able to show by means of SEC‐MALS assays that the addition of 150 mM NaCl to monomeric hLDH‐A enhanced the level of its conversion into the corresponding dimeric and tetrameric forms over what observed in the presence of 25 mM NaCl (Figure 2). Furthermore, similar assays revealed that β‐NADH triggers an almost quantitative conversion of monomeric hLDH‐A into hLDH‐5 (Figure 3). Accordingly, the competence of the redox cofactor in promoting the assembly of hLDH‐5 is by far more effective than the analogous action exerted on monomeric hLDH‐A by the addition of NaCl.

As noted above, by performing SEC‐MALS experiments, we detected secondary peaks containing a polydisperse protein population, consisting of dimeric and monomeric hLDH‐A (Figures 2 and 3). To obtain a quantitative estimate of this polydispersity, we took into account the apparent molecular mass of the species eluting from the column and considered it as the sum of the molecular mass of dimeric and monomeric hLDH‐A, with each mass component normalized to its percentage. By these means, the partition of the secondary peaks into dimeric and monomeric hLDH‐A was determined, showing that the tails of these peaks contained monomeric hLDH‐A up to 70%–80% (Figure S8). Unfortunately, the low intensity of the light scattering that occurs at the late‐eluting regions of the tails of the secondary peaks hampered the analysis of their composition. Nevertheless, we would like to suggest that the proportion of eluted monomeric hLDH‐A is progressively enriched over 70%–80% along the distal parts of these peak tails (Figure S8).

By means of stopped‐flow assays, we found that the addition of β‐NADH to monomeric hLDH‐A triggers the assembly of hLDH‐5 (Figure 4). Quantitatively speaking, this assembly was not complete, as indicated by the larger amplitude of the fluorescence decrease mirroring the oxidation of the redox cofactor taking place in the presence of monomeric hLDH‐A (Figure 4a,c). This indeed implies, in turn, a slightly slower reaction compared to that detected with hLDH‐5. Furthermore, it is important to note the higher recovery of tryptophans' fluorescence observed for hLDH‐5, whose emission increase was more than twofold when compared to that featured by monomeric hLDH‐A induced to assemble into the corresponding tetramer (Figure 4d). Altogether, these stopped‐flow assays suggest that the addition of both β‐NADH and NaCl to solutions containing monomeric hLDH‐A represents the best condition to obtain its conversion into hLDH‐5.

The *K*_D_ of the β‐NADH • hLDH‐5 complex was reported as equal to 0.62 μM (Eszes et al., 1996), a value essentially identical to the final concentration of hLDH in our stopped‐flow assays (i.e., 0.6 μM). According to these values, two to three and four molecules of redox cofactor bind to the tetrameric enzyme when the β‐NADH/hLDH‐5 molar ratio is 2 and 8, respectively. Interestingly, when stopped‐flow assays were carried out to determine the association of β‐NADH to hLDH‐5, the observed rate constant featured its maximum extent when the β‐NADH/hLDH‐5 molar ratio was equal to 2 (Figure 6a), whereas an equimolar ratio did suffice to detect the greatest amplitude of the fluorescence changes triggered by the binding event (Figure 6b). Concerning this point, it has to be mentioned that both the invariance of the observed rate constant and the decrease of the amplitudes determined at β‐NADH/hLDH‐5 molar ratios higher than 2 could be due to the limited time resolution of the stopped‐flow technique. Nevertheless, the binding events detected in the presence of monomeric hLDH‐A did significantly differ when compared to those occurring with hLDH‐5, and in particular, the observed rate constant and the corresponding amplitude of the fluorescence changes attained a maximum at a β‐NADH/hLDH‐A molar ratio equal to 16 and 4, respectively (Figure 6a,b). Specifically, we suggest that this output is diagnostic of a low affinity featured by monomeric hLDH‐A for the redox cofactor, with this affinity being progressively increased (at β‐NADH/hLDH‐A molar ratio higher than 4, see Figure 6a) by means of hLDH‐5 assembly.

In agreement with our previous observations (Stefan et al., 2024, 2026), we have shown here that: (i) the inhibitory action exerted against hLDH‐A by the peptides TH2 and TH3 is strictly dependent on the presence of a free aspartate (Figure 8a); (ii) to antagonize hLDH‐A, the long‐chain dicarboxylates crocetin and hexadecanedioic acid specifically rely on their terminal unesterified carboxylates (Figure 8a). In addition to these observations, we reported that the peptides LCO15 and HL60 are quite effective hLDH‐A inhibitors, featuring rather low IC_50_ values (Figure 8c,d). Remarkably, these peptides share with TH2 and TH3 the QN sequence, and respectively contain β‐Ala and an unusual residue instead of aspartate (Figure 7). Therefore, the antagonist action of LCO15 and HL60 does most likely depend on the QN residues, which could interact with the region of the target spanning amino acids 14–19, as previously shown (Jafary et al., 2019). Surprisingly enough, the LCO15 octapeptide is competent in fully antagonizing hLDH‐A (Figure 8a–c) by interfering with the association to the enzyme of β‐NADH (Figure 8e,f). Moreover, it is interesting to note that this interference does quantitatively account for the inhibition exerted by the LCO15 peptide against the catalytic activity of hLDH‐A. We indeed estimated that the addition of 8 μM LCO15 triggers a 22% decrease of the rate constant coupled to the association of the redox cofactor (Figure 8e,f), and a 29% slow‐down of the steady‐state enzyme activity (Figure 8c). To interpret the antagonism exerted by LCO15, we suggest that the strong inhibitory action of this peptide is linked to its bi‐modular structure, which could confer to this octapeptide the competence in binding two hLDH‐A subunits, generating a catalytically unproductive complex.

Among the different candidate inhibitors of LDH‐A that were tested *in cellulo*, the HL60 peptide and the methyl ester of hexadecanedioic acid induced an increase of lactate synthesis in MCF7 cells, and featured an opposite response to lipofectamine (Figure 9). In particular, the addition of lipofectamine enhanced the action of HL60 (albeit to a rather variable extent) and depressed the performance of the C16 methyl ester, suggesting that the cationic lipid promotes the import of the peptide by MCF7 cells and sequesters the esterified long‐chain dicarboxylate. Concerning the mechanism of action of the C16 methyl ester, we propose that this compound is imported and converted by MCF7 cells into its free dicarboxylate counterpart, which is known to uncouple oxidative phosphorylation via the disruption of the Q‐cycle at complex III (Pavlova et al., 2026). Furthermore, we suggest that this perturbation of the mitochondrial electron transfer chain shifts the energetic metabolism toward the glycolytic pathway, leading, in turn, to high levels of lactate (Figure 9). To further investigate the action of HL60 and the methyl ester of hexadecanedioic acid, and to ascertain if they could indirectly affect the level of lactate produced by MCF7 cells, we tested their effect, if any, on the activity of hLDH‐1 *in vitro*. Interestingly enough, we found that both compounds are devoid of any significant action on hLDH‐1 (Figure S9), with this outcome indicating their full selectivity towards hLDH‐5.

According to the observations reported here, there is a striking difference between the effects induced by the LCO15 peptide *in vitro* and *in cellulo*. Specifically, the limited action featured *in cellulo* by LCO15 is most likely due to a poor import by MCF7 cells of this candidate inhibitor, with lipofectamine significantly improving its performance. Considering the high effectiveness featured by LCO15 *in vitro* (Figure 8), it will be of interest to design variants of this peptide enriched with basic amino acids, which are known to promote the uptake of cell‐penetrating peptides (Gori et al., 2023; Green & Lowenstein, 1988; Joliot et al., 1991). Alternatively, it could be worthwhile to test if the entrapment of the LCO15 peptide in lipidic vesicles would efficiently promote the import of this compound by MCF7 cells.

We recently reported that the long‐chain dicarboxylate crocetin is highly effective against the assembly *in vitro* of hLDH‐5, as substantiated by the IC_50_ value of this action, which was determined as equal to 436 ± 36 nM (Stefan et al., 2026). Remarkably, crocin, that is, the digentiobiose ester of crocetin, was found devoid of any competence in inhibiting *in vitro* the conversion of monomeric hLDH‐A into its tetrameric counterpart (Stefan et al., 2026). It is therefore interesting to note that we confirmed here these observations (Figure 8), and that we obtained an opposite outcome *in cellulo* (Figure 9). These apparently conflicting findings are most likely related to a different capability of MCF7 cells in importing crocetin and crocin, with the ester being largely preferred over the free dicarboxylate. Furthermore, it is reasonable to suppose that the imported crocin is subjected to its conversion into crocetin, which would accordingly represent the active compound. It should indeed be mentioned that when crocetin and crocin were tested on different human cancer cell lines, crocetin was always found more effective than crocin in impairing cells viability (Kim et al., 2014). Importantly enough, crocetin was found to feature an IC_50_ of about 110 μM when tested with A549 and HeLa cancer cell lines, and was fully inactive against nonmalignant human fibroblast lung cells (Granchi et al., 2017). To ascertain the conversion of crocin into crocetin *in cellulo*, we will commit future work to detect crocetin in lysates of MCF7 cells previously exposed only to crocin. Furthermore, considering that high levels of β‐glucosidase 1 are detected in MCF7 cells (Zhou et al., 2017) it will be of interest to test if inhibitors of this enzyme modulate the level of crocetin in MCF7 cells incubated with crocin.

## 4 CONCLUDING REMARKS

To efficiently sustain glycolysis, malignant cells rely on the catalytic action performed by lactate dehydrogenase. In particular, the LDH activity *in cellulo* has been determined as equal to 3.75 fmol/cellper minute in MCF7 cells (Diers et al., 2012). This value translates, by considering the volume of a single MCF7 cell equal to 3500 μm^3^ (Wen et al., 2017), into 17.8 μM/s. This high level of enzyme activity, to which the overexpression of hLDH‐A in malignant cells does significantly contribute, is appropriate to obtain a rapid generation of energy and the metabolic reprogramming necessary for tumor growth.

We have presented here a biochemical characterization of monomeric human LDH‐A, and by means of this analysis different factors promoting the assembly of this protein into its catalytically active tetrameric counterpart were identified, that is, the ionic strength and β‐NADH binding. Moreover, we compared the kinetics of β‐NADH binding by monomeric and tetrameric hLDH‐A, and we were able to determine the molar excess of the redox cofactor necessary to trigger the monomers‐to‐tetramer transition. Considering the importance of hLDH‐5 in the energetic metabolism of cancer cells, we tested peptides and long‐chain dicarboxylates as candidate inhibitors of the assembly of tetrameric hLDH‐A. Overall, we found that cyclic peptides and long‐chain unsaturated dicarboxylates represent appealing tools to tackle this strategy. Therefore, it is our hope that the findings reported here will be of help to obtain further insights to devise an effective approach against the proliferation of malignant cells.

## 5 MATERIALS AND METHODS

### 5.1 Purification of monomeric and tetrameric hLDH‐A

Monomeric hLDH‐A and hLDH‐5 were essentially obtained as previously reported (Stefan et al., 2024). The gene coding for hLDH‐A and optimized for *E. coli* codon usage was cloned into the pET9a expression plasmid using the *Nde*I and *Bam*HI sites. *E. coli* BL21(DE3) electrocompetent cells were then transformed with this construct, and transformants were selected using Petri dishes containing Luria‐Bertani agar medium (LB‐agar) supplemented with kanamycin (40 μg/mL). Single colonies were purified and subsequently picked up to inoculate LB‐kan liquid medium. Pre‐cultures were obtained incubating the inoculated media for 15 h at 37°C under shaking conditions (180 rpm). The pre‐cultures were diluted (1:500) into fresh LB‐kan medium and grown at 30°C for 9 h. Finally, 1 mM isopropyl‐β‐D‐thiogalactopyranoside (IPTG) was added, and the induced cultures were incubated at 30°C for 15 h. The cells subjected to overexpression of hLDH‐A were harvested by centrifugation (4,500 × g, 20 min, 4°C), and the pellets were resuspended in 50 mM Na_2_HPO_4_, 150 mM NaCl, 1 mM ethylenediaminetetraacetic acid, pH 7.5 (buffer A). Proteins were extracted by sonication (Misonix‐3000 sonifier, output level of 18 W for 15 s, followed by a 15 s cooling interval, for 4 cycles), and the crude protein extract accordingly obtained was centrifuged (13,000 × g, 30 min, 4°C), the pellet was discarded, and the supernatant was immediately loaded onto a HiTrap Blue column (5 mL, Cytiva, Marlborough, MA, USA), previously equilibrated with buffer A. After extensive washing of the column with buffer A, LDH‐A was eluted with the same buffer supplemented with 0.5 M NaCl. The best fractions (as determined by activity assays and sodium dodecyl sulphate polyacrylamide gel electrophoresis analysis) were pooled, and solid NaCl was added to increase its concentration to 2 M in the pooled fractions. The sample obtained by these means was loaded onto a HiTrap Phenyl FF column, previously equilibrated with Buffer A containing 2.0 M NaCl. After washing the column, a reverse NaCl gradient (2.0–0 M) was applied (10 column volumes), and afterwards LDH‐A was eluted with buffer A. The best eluted fractions were pooled, concentrated to 2 mL and loaded onto a Superdex‐200 column, equilibrated with: (i) 10 mM Tris–HCl, pH 7.5, to isolate monomeric hLDH‐A; (ii) 10 mM Tris–HCl, 150 mM NaCl, pH 7.5, to obtain hLDH‐5. After pooling the fractions containing homogeneous LDH‐A, aliquots of the concentrated purified enzyme were stored at −20°C until used. Protein concentration was determined according to Bradford (Bradford, 1976). Unless otherwise stated, enzyme concentrations are expressed as total subunits. When necessary, the buffer used to store monomeric hLDH‐A was exchanged using HiTrap desalting columns.

### 5.2 Blue‐native polyacrylamide gel electrophoresis

To perform blue‐native PAGE, 7.5% (w/v) polyacrylamide gels were prepared in 330 mM BisTris buffer (pH 7.3), using a stock solution containing acrylamide/bis‐acrylamide (37.5:1) provided by Bio‐Rad (Hercules, CA, USA). The anodic buffer was 50 mM BisTris (pH 7.3), and the cathodic chamber was filled with 15 mM BisTris, 50 mM 3‐(N‐Morpholino)propanesulfonic acid, 0.02% (w/v) Coomassie Brilliant Blue G250, pH 7.3. Samples were conditioned by dilution in 20 mM BisTris, 6% (v/v) glycerol, 1% (w/v) Coomassie Brilliant Blue G250, pH 7.3. Electrophoresis was started at 170 V for 60 min, and the voltage was subsequently increased to 200 V for 30 min. At the end of the electrophoretic run, gels were rinsed with water, and then stained using standard procedures.

### 5.3 Multi‐angle light scattering

SEC coupled with MALS detection was carried out using a high performance liquid chromatography (HPLC) system equipped with a 515 HPLC pump and a 2487 dual wavelength absorbance detector (both provided by Waters, Sesto San Giovanni, Italy), a Wyatt Dawn Heleos MALS and a Wyatt Optila T‐rEX differential refractive index detector (Wyatt technology, Santa Barbara, CA, USA). Samples (200 μL) were injected in a Superose 12 column (Cytiva, Marlborough, MA, USA), conditioned with the appropriate buffer, and the chromatographic run was performed at a 0.5 mL/min flow rate. Data from SEC‐MALS chromatographic runs were analyzed using ASTRA V software (release 5.3.4.20; Wyatt Technology, Santa Barbara, CA, USA).

### 5.4 Activity assays

The enzyme‐catalyzed reduction of pyruvate was assayed by determining the decrease in absorbance at 340 nm related to the oxidation of the reduced cofactor β‐nicotinamide adenine dinucleotide (β‐NADH). The extinction coefficient of β‐NADH at 340 nm was considered equal to 6.22 × 10^3^/M/cm (Bernofsky & Wanda, 1982). All the assays were performed at 25°C using a Cary 300 Bio spectrophotometer.

The inhibition exerted by peptides or dicarboxylates on LDH‐A activity was tested by mixing 6 nM enzyme with the candidate antagonist in 10 mM Tris–HCl (pH 7.5). To contain the absorbance at 340 nm, crocetin and crocin were used at 20 μM. Reactions were started by the addition of 500 μM pyruvate and 125 μM β‐NADH. hLDH‐1 (1 mg/mL, lot 1002937‐7) was obtained from AbCam (Cambridge, UK).

### 5.5 Stopped‐flow experiments

The kinetics of the binding of β‐NADH to monomeric hLDH‐A was assayed by means of the stopped‐flow technique. All the assays were performed at 20°C in 10 mM Tris–HCl (pH 7.5), using a KinTek SF2004 instrument (KinTek, Snow Shoe, PA, USA) equipped with an observation cell of 0.5 cm path length. The fluorescence of hLDH‐A tryptophanes was determined by exciting samples at 280 nm, and detecting emitted light using a longpass filter (cut‐on wavelength 350 nm). The enzyme syringe was filled with 1.2 μM monomeric hLDH‐A (and eventually with β‐NADH or 16 μM of the LCO15 octapeptide), and the substrate syringe contained β‐NADH (and 1 mM pyruvate when necessary). For each measurement, 5–10 traces were averaged.

### 5.6 Colorimetric assays of lactate secretion by cultured human cells

The inhibitory action of peptides against hLDH‐A in cultured cells was tested by assaying the concentration of lactate released by MCF7 cell cultures. To this aim, cells were seeded in duplicate in 24‐wells plates (2 × 10^5^/well) and let to adhere overnight; the culture medium was then replaced by Krebs‐Ringer buffer (300 μL/well). Stock solutions of peptides (4 or 8 mM in DMSO) were diluted in Krebs‐Ringer buffer to a final concentration of 320 or 640 μM and mixed with an equal volume of 4% (v/v, in Krebs‐Ringer buffer) Lipofectamine 2000 (Invitrogen). The solution accordingly obtained was thoroughly mixed, kept for 25 min at room temperature, and 100 μL were finally added to each sample, obtaining a final peptide concentration equal to 40 or 80 μM. The concentration of lactate released in the Krebs‐Ringer buffer was determined after 4 h of incubation at 37°C, using the procedure previously described (Rossi et al., 2023). Each experiment was performed in triplicate and included control samples consisting of untreated cell cultures exposed to the same final concentration of DMSO (1%, v/v) and Lipofectamine (0.25%, v/v) to which were exposed the treated samples.

### 5.7 Analytical methods

Solvents and reagents were utilized as purchased. Compounds were isolated by semi‐preparative reverse phase high performance liquid chromatography (RP‐HPLC)using an Agilent 1100 series apparatus, equipped with a RP XSelect Peptide CSH C18 OBD column (Waters), 19 × 150 mm, particle size 5 μm, pore size 130 Å; eluent 1:1 H_2_O/CH_3_CN, flow rate 10 mL/min; diode array detector (DAD) 210 or 254 nm. Purities were assessed to be ≥95% by analytical HPLC, using an Agilent 1100 series apparatus, equipped with an RP column Phenomenex C18 mod. 00D‐4439‐Y0‐FA, 100 × 3.0 mm, particle size 3 μm, pore size of 110 Å; the eluent gradient was from 9:1 H_2_O/CH_3_CN to 2:8 H_2_O/CH_3_CN in 20 min, flow rate 1.0 mL/min; DAD 254 nm. Electrospray ionization mass spectroscopy (ESIMS) analysis was performed on a single quadrupole HP 1100 MSD detector (Agilent), with a drying gas flow of 12.5 L/min. Nuclear magnetic resonance (NMR) spectroscopy was performed with a Bruker 600, at 600 MHz for ^1^H NMR, 150 MHz for ^13^C NMR, in 5 mm tubes at 298 K. Chemical shifts are standardized on CDCl_3_ (7.26 ppm for ^1^H NMR and 77.16 ppm for ^13^C NMR), or (CD_3_)_2_SO (2.50 ppm for ^1^H NMR, 39.52 ppm for ^13^C NMR). ^1^H NMR resonances were attributed after 2D gCOSY.

### 5.8 Solid‐phase synthesis of the linear peptide precursors

To achieve the loading of the C‐terminal amino acid onto 2‐chlorotrityl chloride (2‐CTC) resin, this polymeric material (0.4 g, 100–300 mesh, loading: 1.2 mmol/g) was placed in a polypropylene syringe fitted with a polyethylene porous disk. The resin was then swollen in anhydrous dichloromethane (DCM) for 15 min. A solution of Fmoc‐Gln(Trt)‐OH (0.3 g, 0.5 mmol) and *N*,*N*‐diisopropylethylamine (DIPEA, 0.09 mL, 0.5 mmol) in DCM (5 mL) was added, and the mixture was shaken for 5 min. An additional 1.5 eq. of DIPEA (0.14 mL, 0.75 mmol) was then added and shaking was continued for 1 h at room temperature (RT). Capping was performed by adding HPLC‐grade MeOH (0.5 mL) to the suspension and shaking for 30 min. The resin was then filtered and washed sequentially with DCM (3×), MeOH (3×), and dimethylformamide (DMF, 3x).

The Fmoc protecting group was removed by treating the resin with a 20% (v/v) solution of piperidine in DMF (5 mL) while shaking at RT for 20 min. The process was repeated once after an initial DMF wash. The resin was then filtered and washed thoroughly with DCM, MeOH, and DMF (3 × 5 mL each).

To obtain peptide bond formation, a mixture of Fmoc‐protected amino acid (0.4 mmol) and 1‐hydroxybenzotriazole (HOBt, 0.4 mmol) in 1.1 DCM/DMF (5 mL) was shaken for 10 min. This solution was then added to the pre‐swollen aminoacyl‐resin, followed by the addition of *N*,*N*′‐dicyclohexylcarbodiimide (DCC, 0.5 mmol) under N_2_ atmosphere. The mixture was shaken for 2 h at RT, then the resin was filtered and washed thoroughly with DCM, MeOH, and DMF (3 × 5 mL each). Coupling efficiency was monitored using the Kaiser test.

The linear peptides were cleaved from the resin support using a mixture of 0.5% trifluoroacetic acid (TFA) in DCM (10 mL) for 1 h at RT. The mixture was filtered, and the resin was washed with 5% TFA in Et_2_O (3 × 10 mL). The combined filtrates were concentrated at reduced pressure. The crude peptide was precipitated as a TFA salt by adding ice‐cold Et_2_O, collected by centrifugation, and triturated with cold Et_2_O.

To obtain Fmoc‐Aba, a stirred solution of Aba (2 g; 13.23 mmol) in 1:1 dioxane/H_2_O (10 mL) at 0°C, Na_2_CO_3_ (4.19 g; 39.7 mmol) and Fmoc‐Cl (3.59 g; 13.23 mmol) were added, and the mixture was slowly allowed to reach RT. After 12 h, the reaction mixture was cooled to 0°C and 1*N* HCl was added to adjust pH to 5. The precipitated solid which formed was filtered, washed with H_2_O and hexane (5 mL), and dried under reduced pressure to afford the product (4.4 g; 11.78 mmol, 90%) as a white solid. ESI MS *m*/*z* expected for [C_23_H_20_NO_4_]^+^ 374.1, found 396.1 [M + Na]^+^.

Analytical characterization of the linear peptides: (i) H‐Asn‐β‐Ala‐Gly‐Gln(Trt)‐OH (vv). ESI MS m/expected for [C_36_H_44_N_7_O_8_]^+^ 702.3, observed 702.3 [M + H]^+^; (ii) H‐(Asn‐β‐Ala‐Gly‐Gln(Trt))_2_‐OH (nn). ESI MS *m*/*z* expected for [C_72_H_85_N_14_O_15_]^+^ 1385.6, observed 1385.7 [M + H]^+^; (iii) H‐(Asn‐Aba‐Gln(Trt))_2_‐OH (uu) (Aba, 4‐(aminomethyl) benzoic acid). ESI MS *m*/*z* expected for [C_72_H_73_N_10_O_11_]^+^ 1253.5, observed 1275.5 [M + Na]^+^.

### 5.9 Cyclization of peptides under pseudo‐high dilution conditions

Pseudo high‐dilution cyclization was attained using a KD Scientific single infusion syringe pump equipped with a 10 mL glass syringe. The linear precursors were utilized in the cyclization step without further purification. A solution of the linear peptide (0.15 mmol) in DMF (10 mL) was added via the syringe pump over 16 h to a stirred solution of hexafluorophosphate benzotriazole tetramethyl uronium (0.45 mmol), HOBt (0.45 mmol), and DIPEA (0.9 mmol) in DMF at RT. Following completion of the addition, the reaction was stirred for an additional 2 h. Then the solvent was removed under reduced pressure, and the crude cyclic peptide was isolated by semi‐preparative RP‐HPLC on a C18 column.

### 5.10 Trt group deprotection

Removal of the acid‐labile Trt side‐chain protecting groups was achieved by treatment with a cleavage cocktail consisting of TFA/thioanisole/water/EDT/phenol (20:1:1:0.5:0.5 v/v, 10 mL) for 4 h at RT. The final peptide products were obtained in quantitative yield. The peptides were isolated by crystallization in MeOH/Et_2_O. Purities were confirmed to be >95% by analytical RP‐HPLC. Peptide identity was confirmed by ESI MS and NMR spectroscopy in DMSO‐*d*_6_.

### 5.11 Synthesis of dimethyl hexadecanoate

Hexadecanedioic acid (62 mg, 0.2 mmol) was dissolved in anhydrous methanol (10 mL) in a round‐bottom flask. The solution was cooled in an ice bath, followed by the dropwise addition of thionyl chloride (143 mg, 1.2 mmol). The reaction mixture was stirred at 0°C for 2 h, with progress monitored by thin‐layer chromatography (TLC). Upon total consumption of the starting material, the reaction was quenched. The solvent was removed under a stream of compressed air (or by means of reduced pressure). The resulting residue was treated with an aqueous basic solution and extracted with ethyl acetate (EA). The organic phase was washed with acidic water until a neutral pH was achieved. The combined organic layers were concentrated via rotary evaporation to afford the crude product. Purification was performed using flash column chromatography (eluent: EA/cyclohexane 1:20 v/v) giving a white solid (63 mg, 93%).

### 5.12 Analytical features of peptides and long‐chain dicarboxylates

c[(Asn‐β‐Ala‐Gly‐Gln)_2_] (LCO15): ^1^H NMR (600 MHz, DMSO‐*d*_6_) *δ* 8.55 (br.s, 2H), 8.11 (br.s, 4H), 7.05–7.20 (m, 6H), 6.82 (br.s, 2H), 6.76 (br.s, 2H), 4.54–4.33 (m, 2H), 4.04–3.93 (m, 2H), 3.92–3.85 (m, 2H), 3.60–3.70 (m, 2H), 3.10–3.20 (m, 2H), 2.68–2.58 (m, 2H), 2.25–2.45 (m, 6H), 2.23–2.02 (m, 4H), 1.96–1.65 (m, 4H). ^13^C NMR (151 MHz, DMSO‐*d*_6_) *δ* 26.53, 31.19, 34.81, 35.81, 170.42, 170.63, 170.80, 171.19, 172.19, 173.65 (Figure S10). ESI MS *m*/*z* expected for [C_28_H_45_N_12_O_12_]^+^ 741.3, found 741.3 [M + H]^+^.

c[(Asn‐Aba‐Gln(Trt))_2_] (HL60): ^1^H NMR (600 MHz, DMSO‐*d*_6_) *δ* 8.92 (d, *J* = 5.7 Hz, 2H), 8.25 (d, *J* = 8.5 Hz, 2H), 7.96 (dd, *J* = 8.2, 3.8 Hz, 2H), 7.47 (d, *J* = 8.2 Hz, 4H), 7.18 (d, *J* = 8.1 Hz, 4H), 6.90 (s, 2H), 6.85 (s, 2H), 4.73 (dd, *J* = 15.6, 8.2 Hz, 2H), 4.55 (dt, *J* = 8.5, 6.1 Hz, 2H), 4.18–4.07 (m, 2H), 3.96 (dd, *J* = 15.7, 3.7 Hz, 2H), 2.62 (dt, *J* = 5.8, 1.9 Hz, 4H), 2.24 (t, *J* = 7.3 Hz, 4H), 1.94 (q, *J* = 7.4 Hz, 4H). ^13^C NMR (151 MHz, DMSO‐*d*_6_) *δ* 26.35, 31.63, 35.75, 41.91, 49.76, 55.74, 126.76, 127.47, 131.88, 142.69, 167.46, 170.41, 171.64, 172.42, 174.28 (Figure S11). ESI MS *m*/*z* expected for [C_34_H_43_N_10_O_10_]^+^ 751.3, found 751.2 [M + H]^+^, 773.2 [M + Na]^+^.

Dimethyl hexadecanedioate (HL62): ^1^H NMR (600 MHz, CDCl_3_) *δ* 3.66 (s, 6H), 2.30 (t, *J* = 7.6 Hz, 4H), 1.55–1.65 (m, 4H), 1.34–1.21 (m, 20H); see Figure S12. ESI MS *m*/*z* expected for [C_18_H_35_O_4_]^+^ 315.2, found 337.2 [M + Na]^+^.

## AUTHOR CONTRIBUTIONS

**Alessandra Stefan:** Investigation. **Luca Gentilucci:** Conceptualization; data curation; supervision; investigation. **Alberto Giuseppe Barbiroli:** Conceptualization; investigation; data curation; supervision. **Valentina Rossi:** Investigation. **Hang Liao:** Investigation. **Giuseppina Di Stefano:** Investigation. **Tingting He:** Investigation. **Alejandro Hochkoeppler:** Conceptualization; investigation; data curation; supervision; writing – original draft; writing – review and editing.

## Supporting information


**Data S1.** Supporting Information.

## Data Availability

The data that support the findings of this study are available on request from the corresponding author. The data are not publicly available due to privacy or ethical restrictions.
