# Supplementary material for: The assembly of tetrameric human L‐lactate dehydrogenase is regulated by ionic strength, β‐NADH binding, cyclic peptides, and long‐chain dicarboxylates
Source: Protein Sci. 2026 Sep 18;35(10):e70796. doi: 10.1002/pro.70796 (PMC13587144; doi:10.1002/pro.70796)
Supplement: Supplementary file 1 [file PRO-35-e70796-s001.pdf]

Supporting information for:

The assembly of tetrameric human L-lactate dehydrogenase is regulated by ionic strength, β-NADH binding, cyclic peptides, and long-chain dicarboxylates

Alessandra Stefan[°$*], Luca Gentilucci[£], Alberto Barbiroli[#], Valentina Rossi[§], Hang Liao[£], Giuseppina Di Stefano[§], Tingting He[£], Alejandro Hochkoeppler[°$*]

[°]Department of Pharmacy and Biotechnology, University of Bologna, Via Gobetti 87, 40129 Bologna (Italy)

[$]CSGI, University of Firenze, Via della Lastruccia 3, 50019 Sesto Fiorentino, FI (Italy)

[£]Department of Chemistry "Giacomo Ciamician", University of Bologna, Via Gobetti 85, 40129 Bologna (Italy)

[#]Department of Food Science, Nutrition and Environment, University of Milano, Via Celoria 2, 20133 Milano (Italy)

[§]Department of Medical and Surgical Sciences, University of Bologna, Via San Giacomo 14, 40126 Bologna (Italy)

[*]Corresponding Author. e-mail: a.hochkoeppler@unibo.it

Table of contents:

Figures S1-S12 pS2-S13

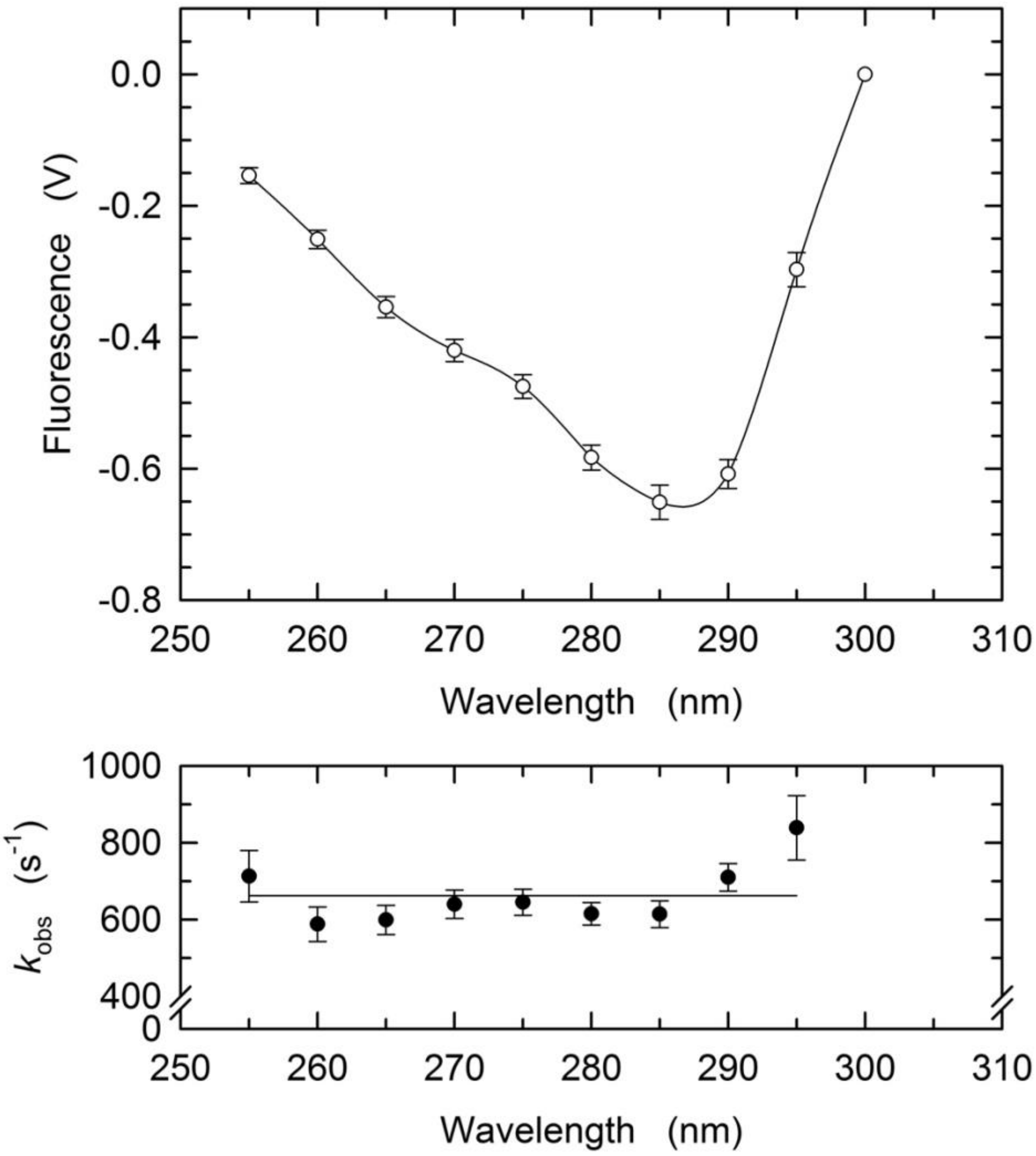

Figure S1. Amplitudes and corresponding rate constants of the fluorescence changes of hLDH-A tryptophanes induced by β-NADH binding and determined as a function of excitation wavelength.

Stopped-flow assays were performed at 20 °C by filling the enzyme syringe with 1.2 µM tetrameric hLDH-A and the cofactor syringe with 19.2 µM β-NADH. Both enzyme and cofactor were buffered with 10 mM Tris-HCl, pH 7.5. To determine the fluorescence quenching of enzyme tryptophanes induced by the binding of β-NADH, samples were excited at the indicated wavelengths, and the emission was detected using a longpass filter. The amplitudes and rate constants (upper and lower panel, respectively) of the detected fluorescence changes were determined by fitting a single exponential equation to the experimental observations. The error bars represent the standard deviation associated to each fitting.

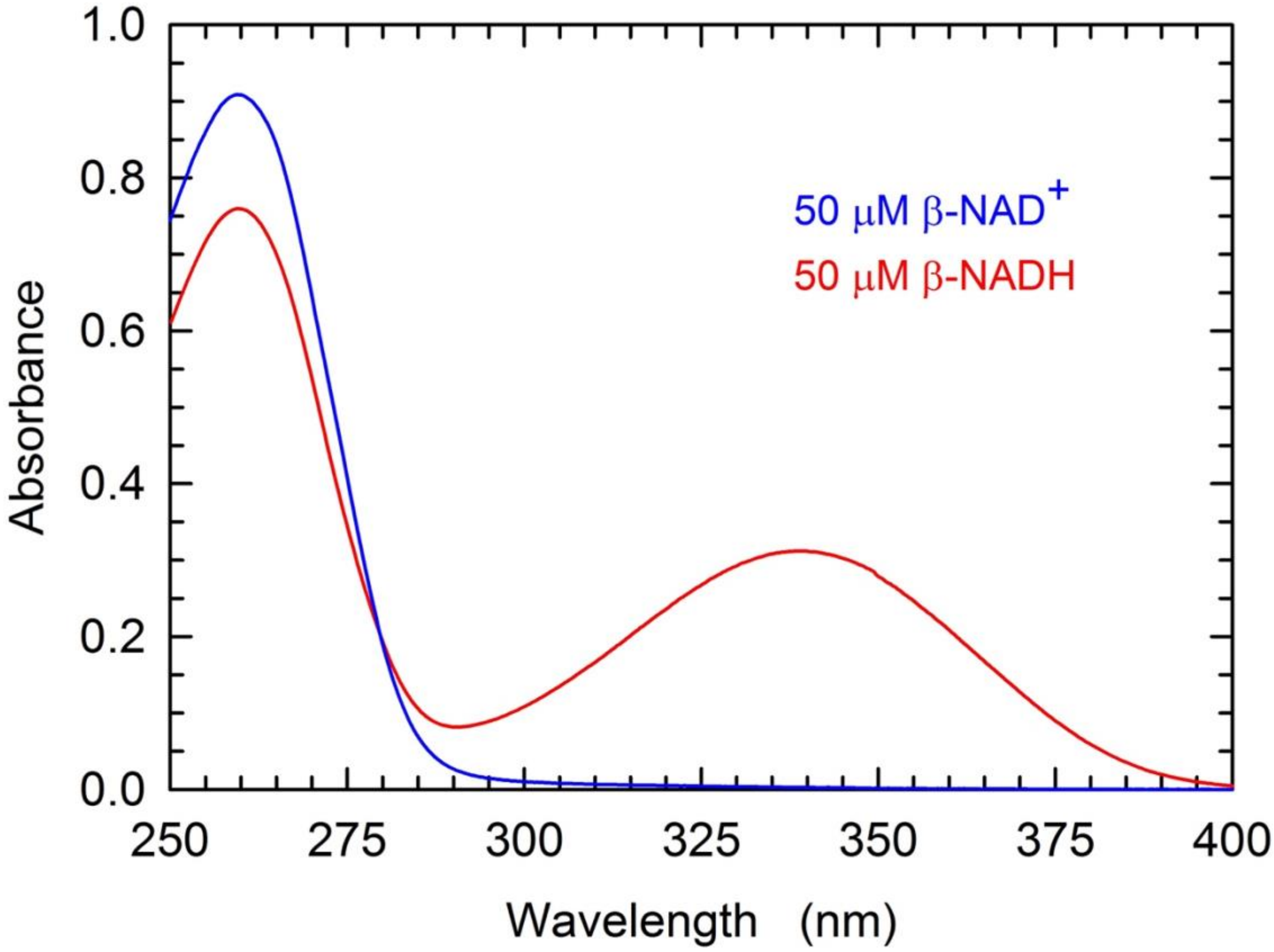

Figure S2. Absorption spectra of β-NADH and β-NAD$^+$.
The absorption spectra of β-NADH and β-NAD$^+$ at 50 μM in 10 mM Tris-HCl (pH 7.5) were obtained using a Cary 300 Bio spectrophotometer and a quartz cuvette featuring a path length equal to 1 cm. The scan rate was equal to 100 nm/min, and the slit width was set to 1 nm.

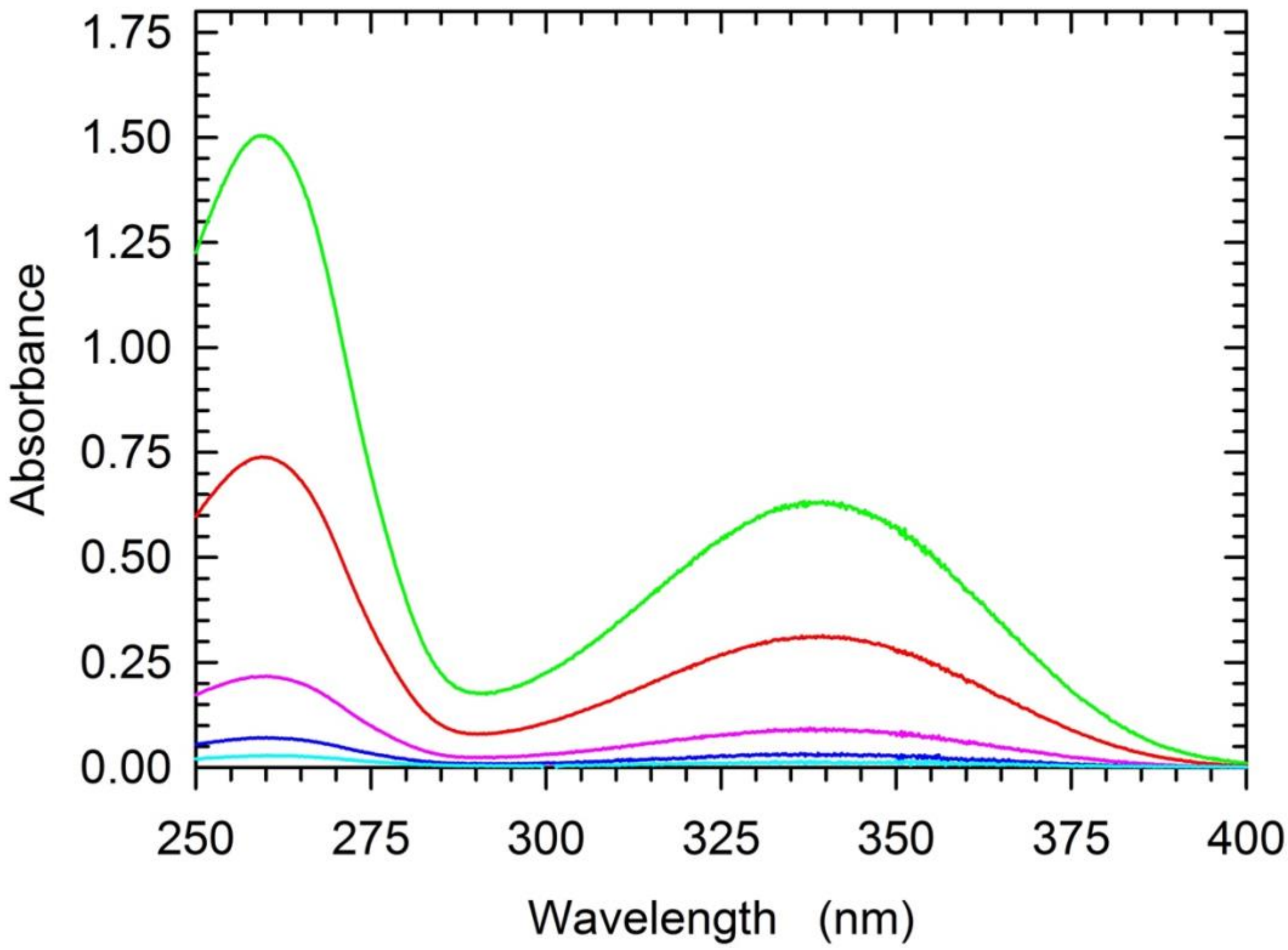

Figure S3. Absorption spectra of samples obtained by ultrafiltration of a solution containing hLDH-5 and β-NADH.
A solution containing 11 µM hLDH-5 and 100 µM β-NADH in 50 mM Tris-HCl, 150 mM NaCl (pH 7.5) was loaded onto an Amicon Ultra ultrafiltration disposable cell (100 kDa M_r cutoff). The ultrafiltration cell was then centrifuged (14,000 x g, 0.3 min) and the absorption spectrum of the eluate (250 µL) obtained by this means is shown with a green line. The retentate (250 µL) was then diluted 1:2, centrifuged (14,000 x g, 0.5 min), and the absorption spectrum of the eluate (400 µL) accordingly obtained is shown with a red line. The ultrafiltration procedure was repeated three times diluting 1:5 the retentate, and the absorption spectra of the ultrafiltrated samples are reported with magenta, blue, and cyan lines, respectively. The last retentate obtained was then used to record its fluorescence excitation spectrum (see Supplementary Figure S4).

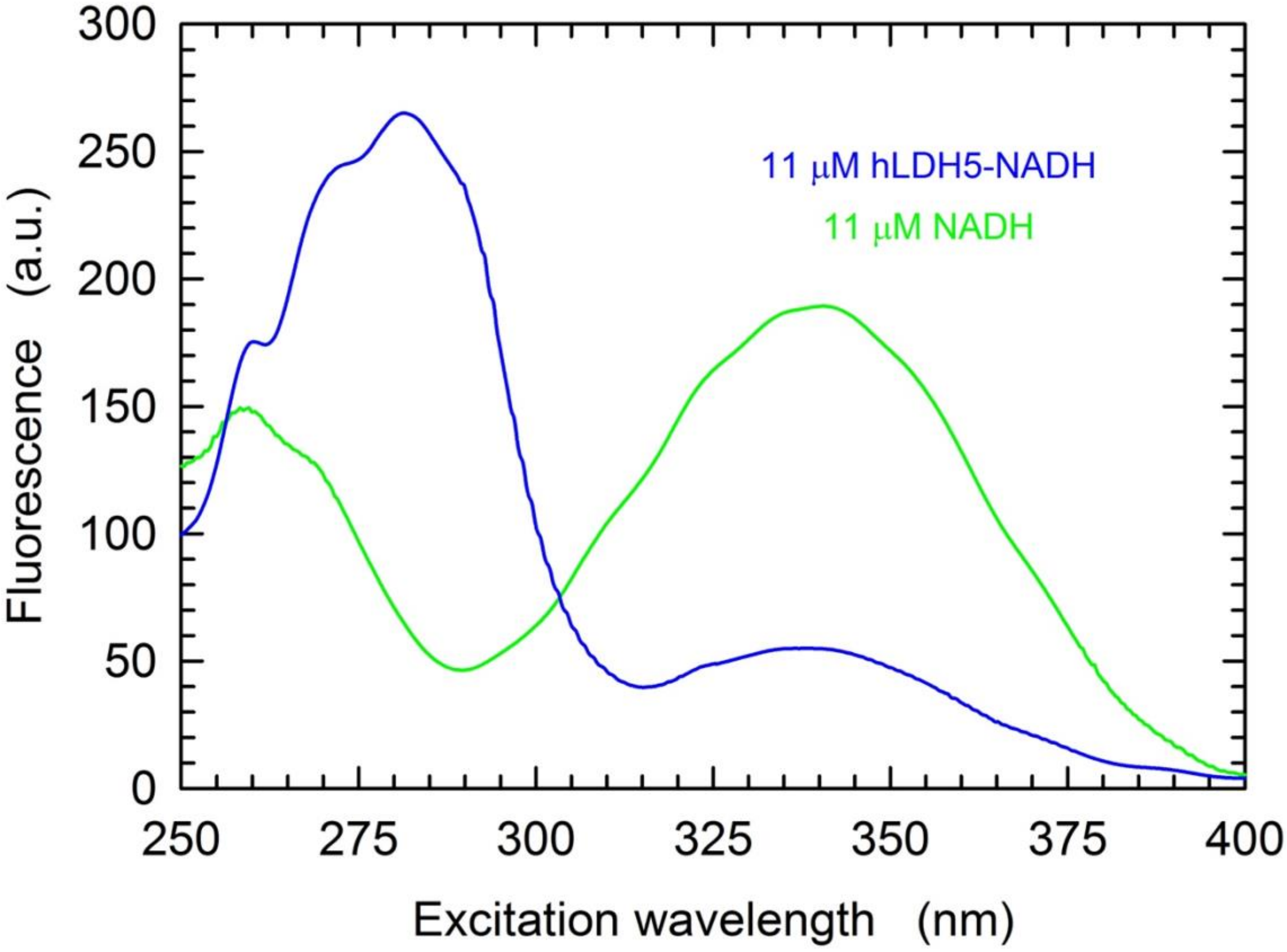

Figure S4. Excitation spectrum of free and hLDH5-bound β-NADH.
The excitation spectrum of 11 μM free and hLDH5-bound β-NADH is shown with green and blue lines, respectively. Both samples were buffered at pH 7.5 with 50 mM Tris-HCl, 150 mM NaCl. Ten spectra were recorded for each sample at 50 nm/min, slits were at 5 nm, gain setting was at 800 V, and the mean is reported.

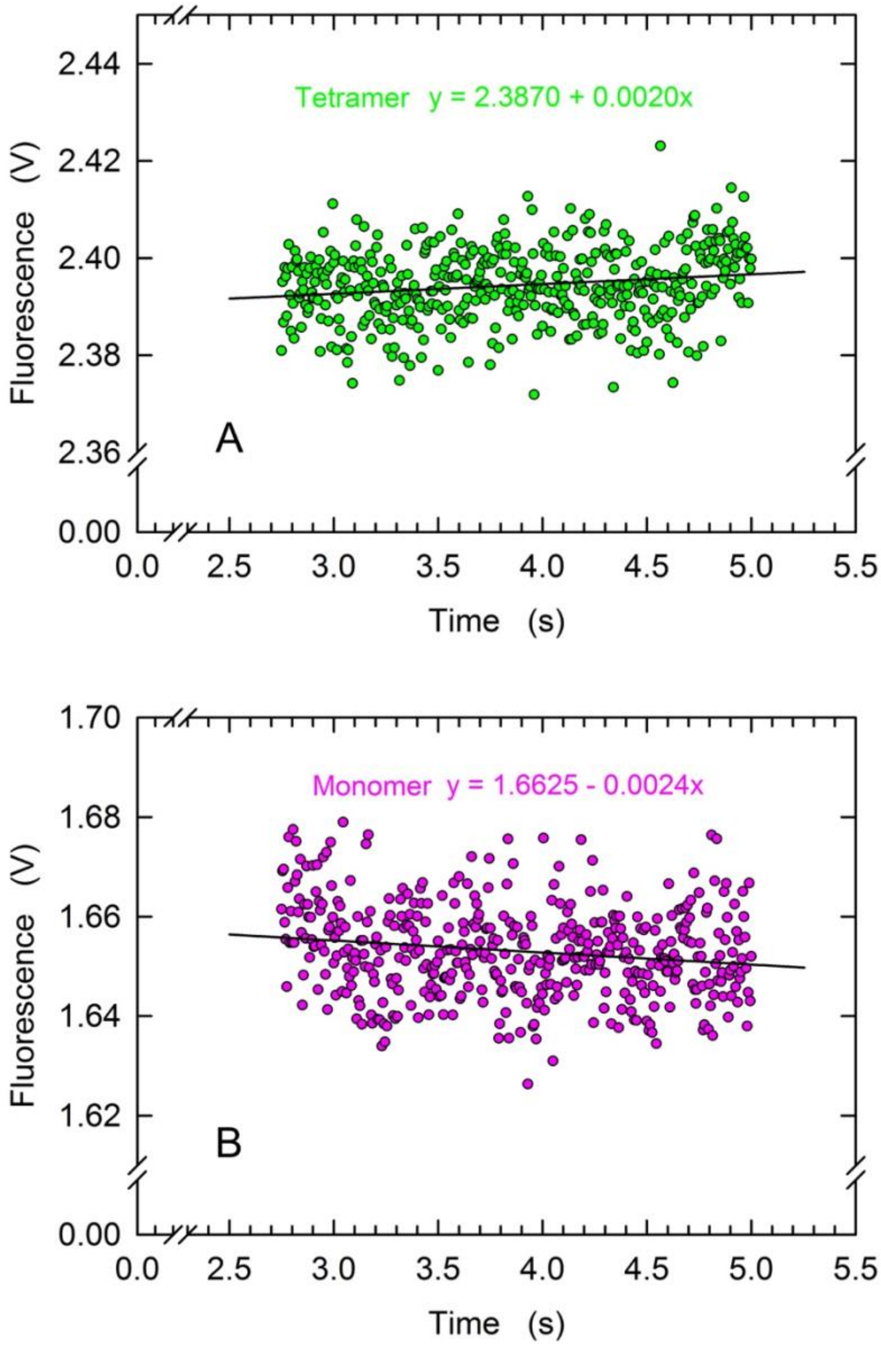

Figure S5. Kinetics of β-NADH oxidation catalyzed by tetrameric and monomeric hLDH-A. (A,B) Late time-course of the fluorescence changes occurring during the β-NADH oxidation linked to the reduction of pyruvate to L-lactate and catalyzed by tetrameric (A) and monomeric (B) hLDH-A. The data reported here are a detail of those shown in Figure 4A (see main text). The continuous lines represent the best fit of a linear equation to the experimental observations.

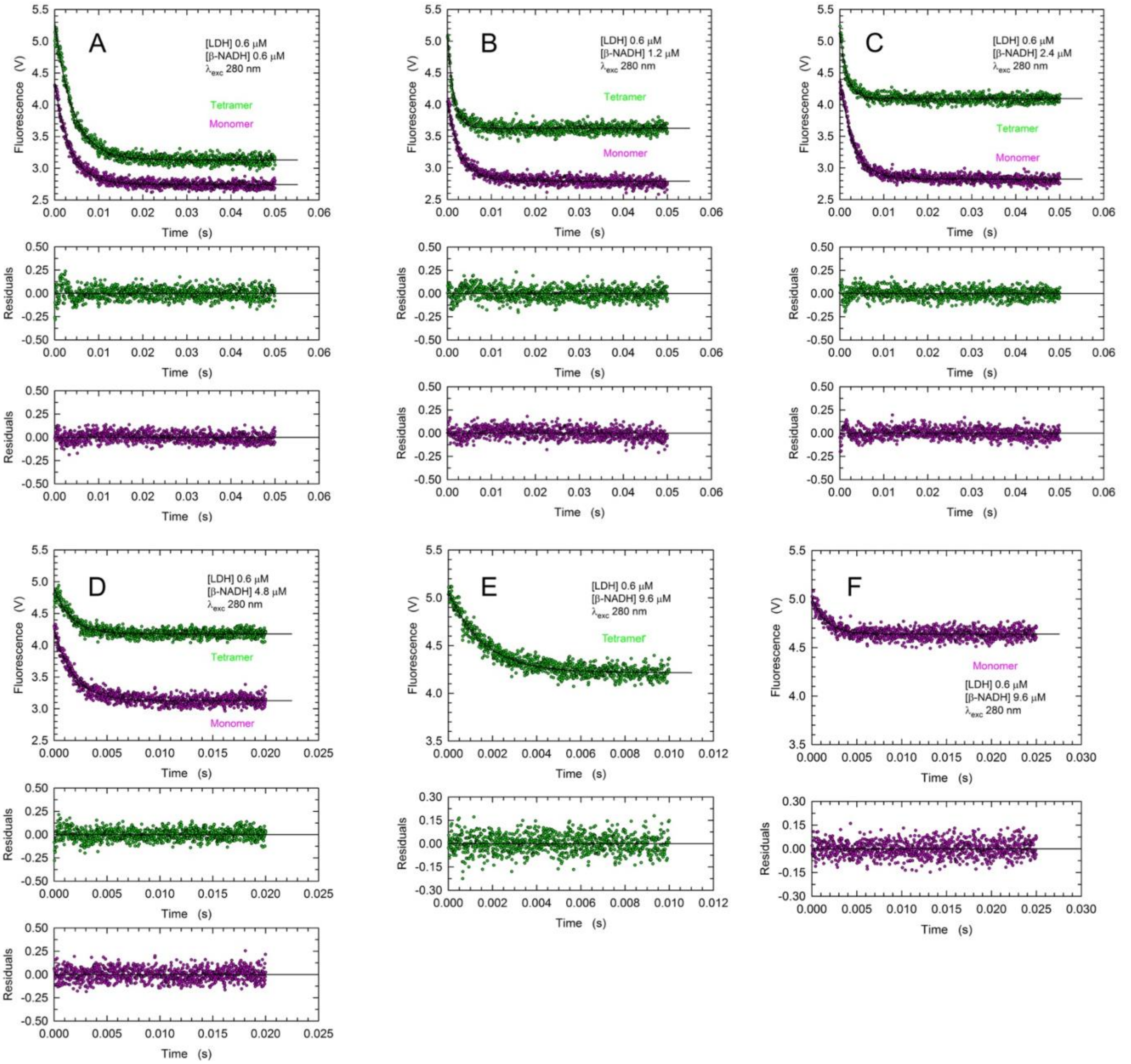

Figure S6. Kinetics of the fluorescence changes (observed over 50, 25, 20, or 10 ms) induced by mixing 1.2 μM human muscle lactate dehydrogenase with β-NADH at different concentrations. (A-F) Stopped-flow assays were performed at 20 °C by filling the enzyme syringe with 1.2 μM tetrameric (green circles) or monomeric (magenta circles) hLDH-A and the cofactor syringe with β-NADH at concentrations ranging from 1.2 to 19.2 μM. Both enzyme and cofactor were buffered with 10 mM Tris-HCl, pH 7.5, and their final concentrations obtained after mixing were as indicated (A-F). To determine the fluorescence of enzyme tryptophanes, samples were excited at 280 nm, and the emission was detected using a longpass filter. The continuous lines represent the best fit of a single exponential equation to the experimental observations.

[β-NADH]/[E]	Enzyme	Observation time (ms)	k_{obs} (s^{-1})	Amplitude (V)
1	Tetramer	50	241±2	2.16±0.01
1	Monomer	50	284±3	1.60±0.01
2	Tetramer	50	714±17	1.49±0.03
2	Monomer	50	332±6	1.27±0.01
4	Tetramer	50	628±16	1.08±0.02
4	Monomer	50	324±4	1.60±0.01
8	Tetramer	20	623±18	0.72±0.01
8	Monomer	20	559±11	1.13±0.01
16	Tetramer	10	633±13	0.85±0.01
16	Monomer	25	639±33	0.37±0.01

Figure S7. Observed rate constants and amplitudes determined by fitting a single exponential equation to the experimental observations shown in Supplementary Figure S6.

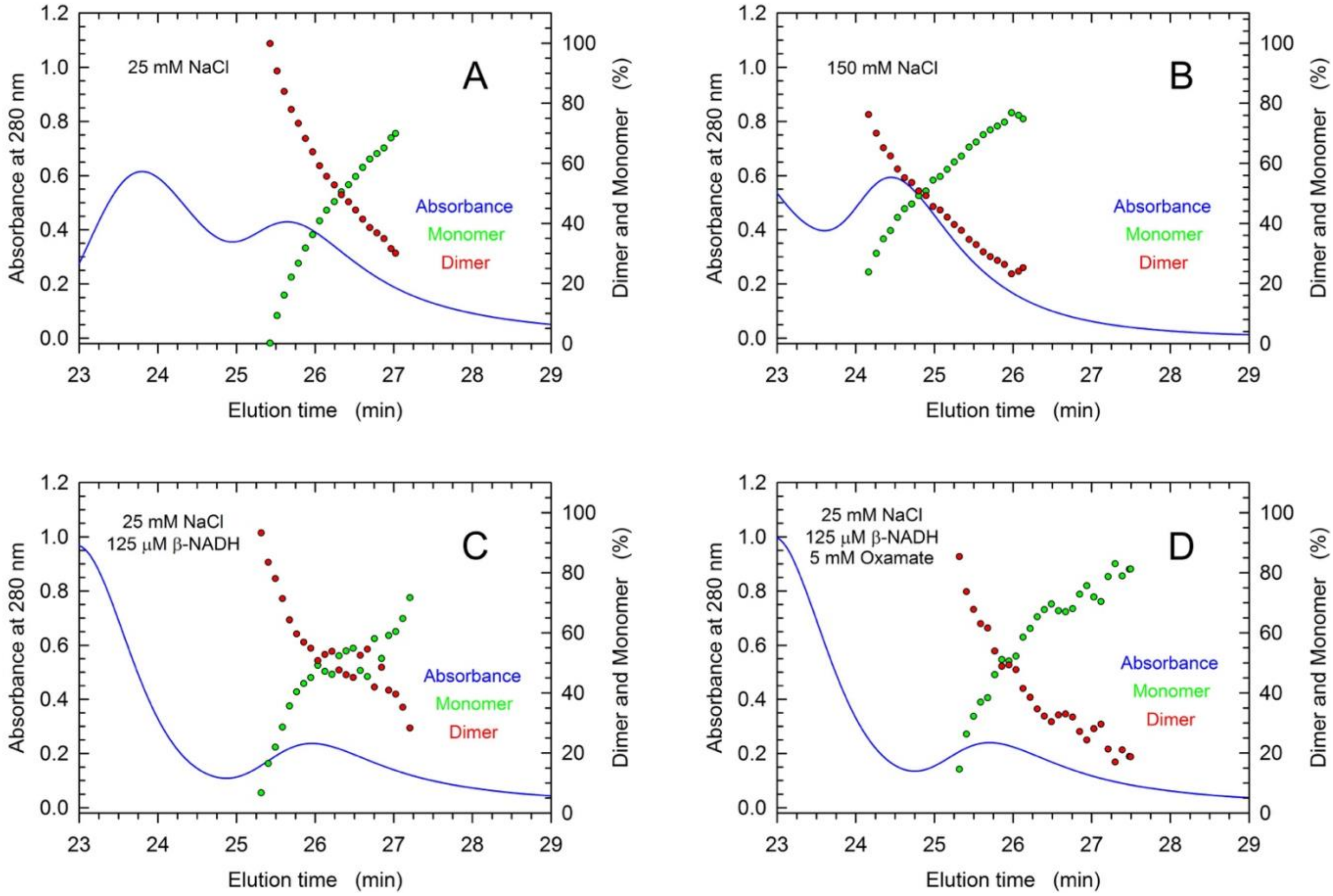

Figure S8. Partition into dimeric and monomeric hLDH-A of the secondary peaks detected by means of SEC-MALS experiments.

(A-D) Details of different chromatograms reporting the detected Absorbance (blue line), and the estimated percentage of dimeric (red circles) and monomeric (green circles) hLDH-A present in each observed peak. The secondary peaks of samples subjected to size exclusion chromatography in the presence of 25 or 150 mM NaCl (A and B, respectively), 25 mM NaCl and 125 μM β-NADH (C), 25 mM NaCl, 125 μM β-NADH, and 5 mM oxamate (D), are shown. The chromatograms to which these details refer are shown in Figures 2 and 3 of the main text.

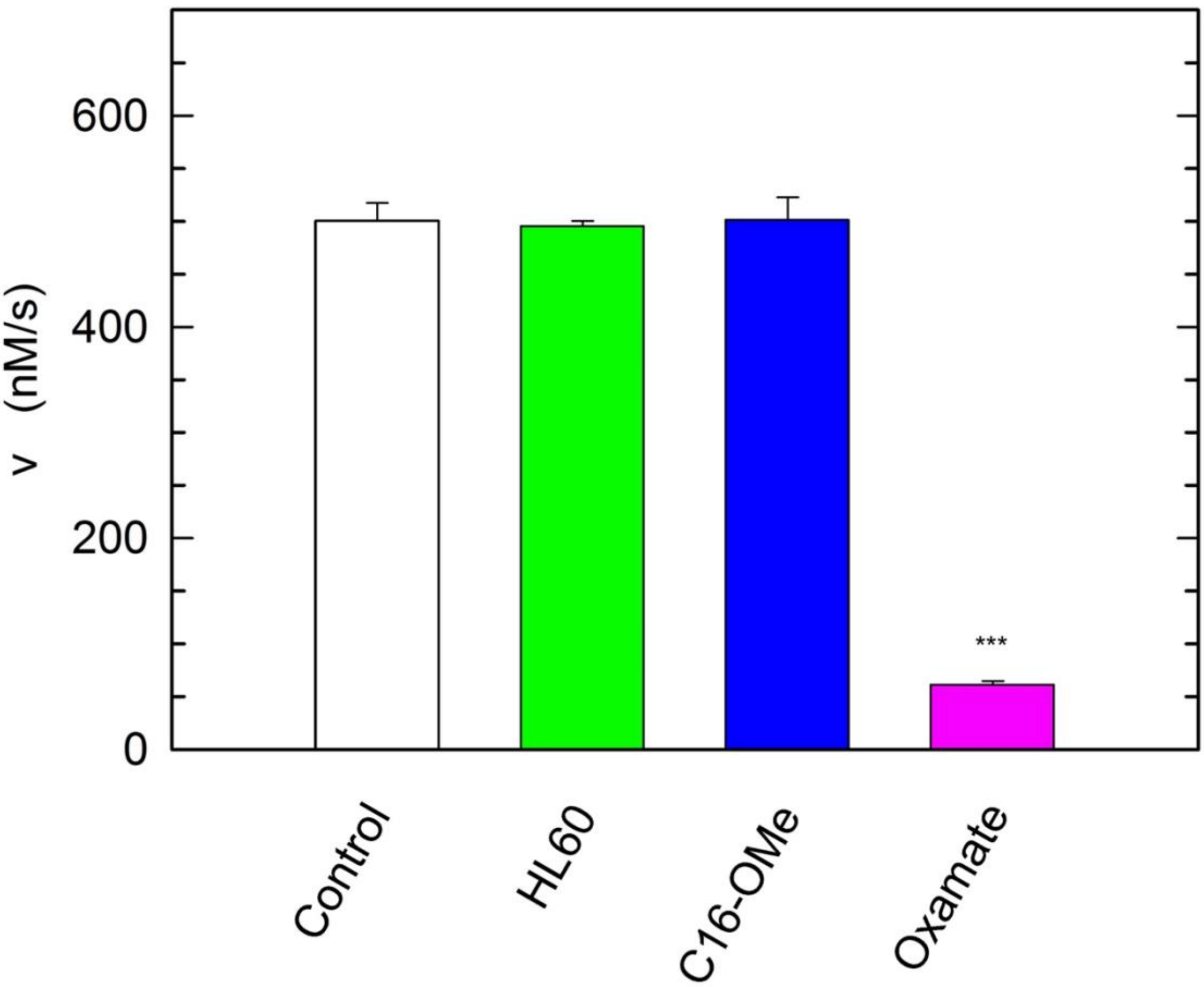

Figure S9. Effect of different compounds on the catalytic action of hLDH-1.
Activity of hLDH-1 (3 nM) assayed at the expense of 500 μM pyruvate and 125 μM β-NADH at pH 7.5 (50 mM Tris-HCl), in the absence (Control) or in the presence of 80 μM HL60, hexadecanedioic acid methyl ester, and oxamate (green, blue, and magenta bars, respectively). The error bars represent standard deviation (n = 3). The experimental observations were compared by the Student's *t*-test. The *** symbol represents a *p* value lower than 0.001.

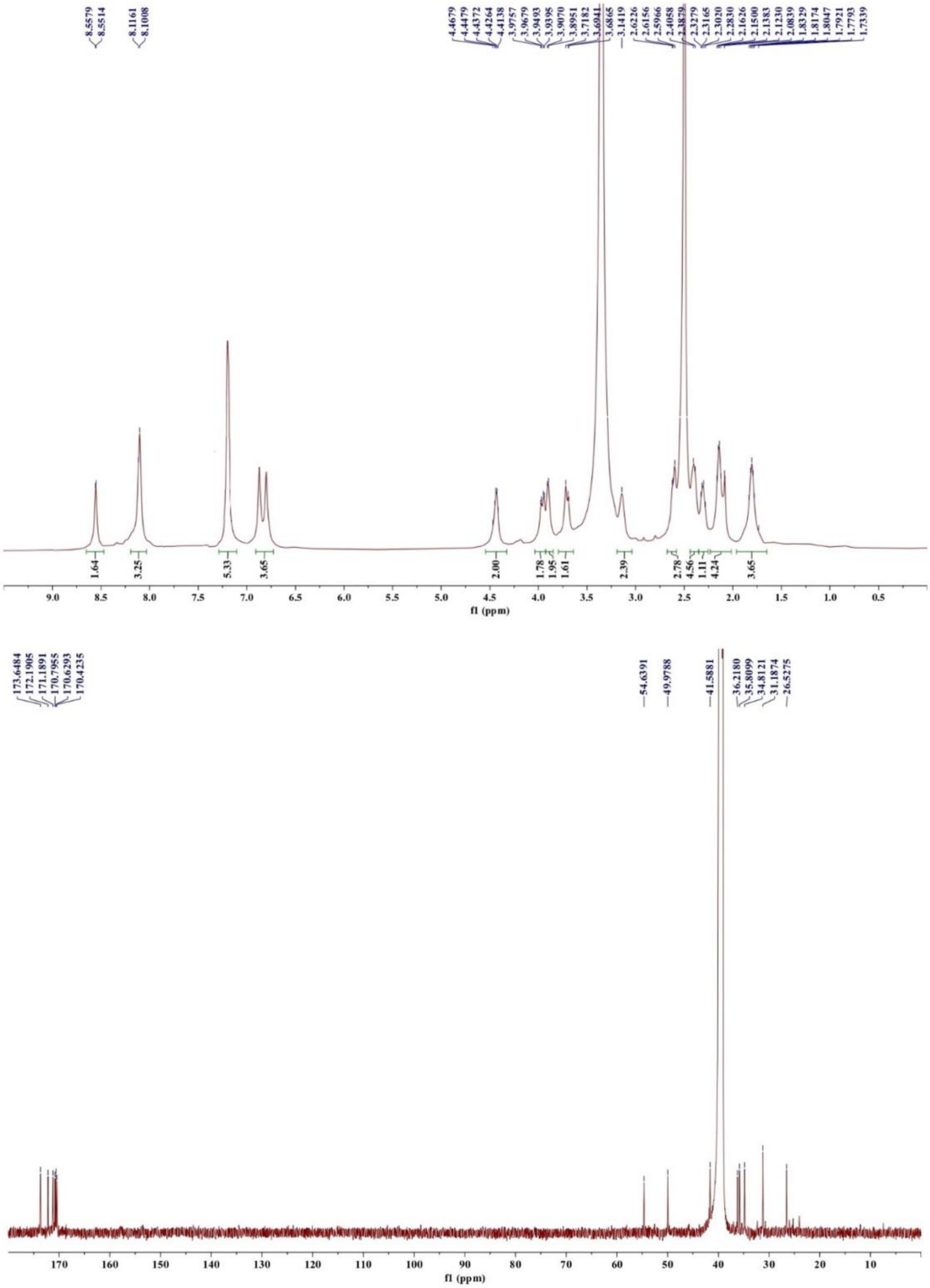

Figure S10. NMR spectra of the purified cyclic LCO15 peptide.

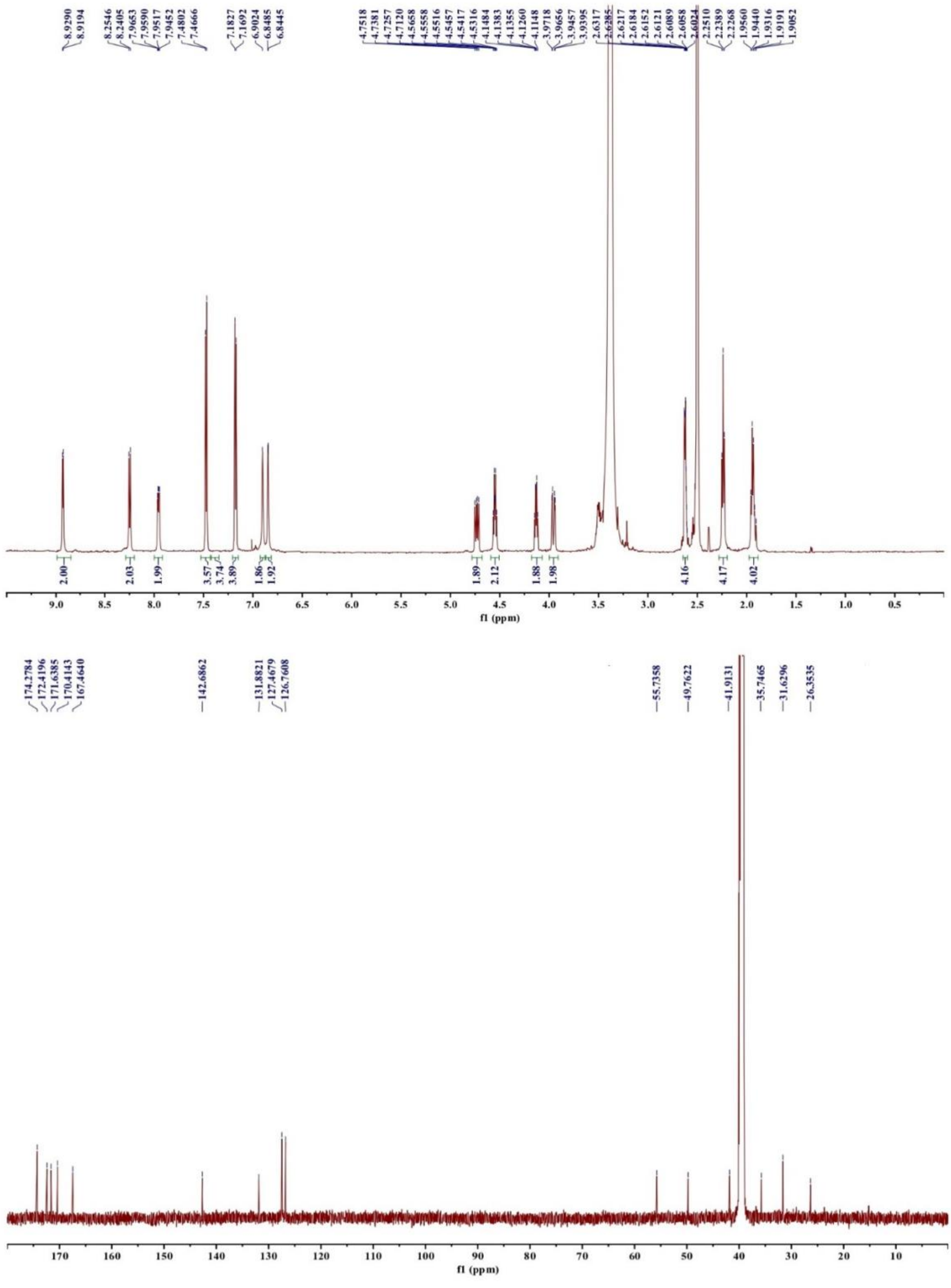

Figure S11. NMR spectra of the purified cyclic HL60 peptide.

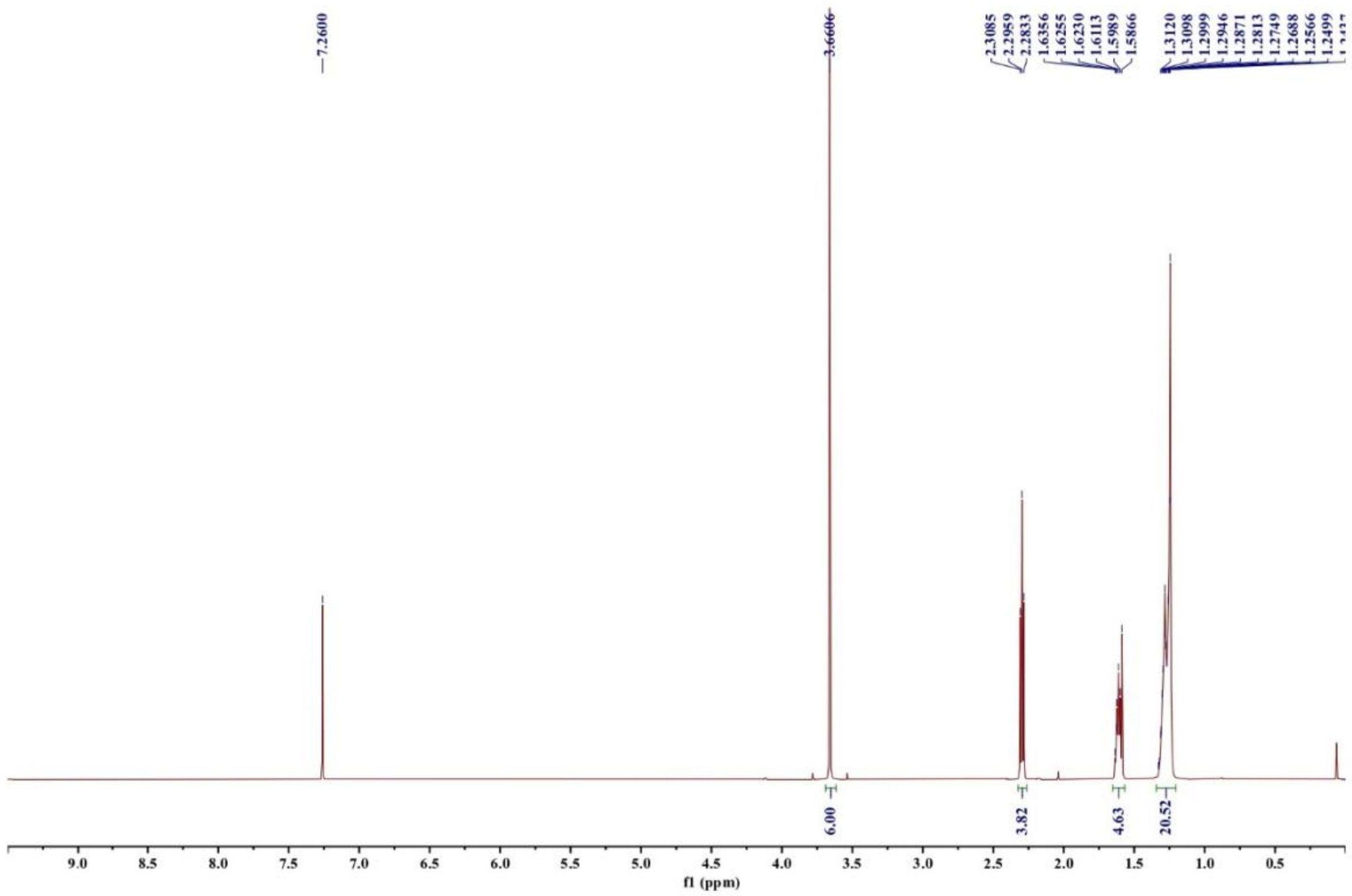

Figure S12. NMR spectrum of purified dimethyl hexadecanedioate.
